# Reciprocal regulation in social support interactions between bereaved parents and their potential supporters: a qualitative study

**DOI:** 10.3389/fpubh.2025.1659628

**Published:** 2025-10-21

**Authors:** Josephine Tognela, Lauren J. Breen, Daniel Rudaizky

**Affiliations:** ^1^School of Population Health, Curtin University, Perth, WA, Australia; ^2^School of Population Health and Curtin enAble Institute, Curtin University, Perth, WA, Australia; ^3^School of Psychological Science, University of Western Australia, Perth, WA, Australia

**Keywords:** grief, bereaved parents, informal social support, reciprocal regulation, qualitative research

## Abstract

**Introduction:**

The death of a child represents one of life’s most profound stressors, often resulting in long-term emotional dysregulation and the potential for mental health diagnoses. This qualitative study explores how bereaved parents experience informal social support attempts.

**Methods:**

Sixteen bereaved parents in Australia were recruited through social media and bereavement support networks and participated in in-depth, semi-structured interviews. Reflexive thematic analysis was applied to interpret participant narratives, with data collection and analysis conducted iteratively. Findings revealed that potential support interactions were rarely neutral: they either offered grounding through perceived safety, or heightened distress through judgement or avoidance.

**Results:**

Four overarching themes were developed: *Societal Norms (The Western World),* articulating societal bereavement norms; *Bereaved Parents’ Experiences (The Untethered World)*, describing bereaved parents’ internal disruption of identity and coherence; *Potential Support Providers’ Perceived Experience (The Uncertain World)*, capturing perceptions of informal social support providers’ uncertainty with providing support; and *Quality of Interactions (The Precarious World)*, detailing how support interactions either alleviated or exacerbated bereaved parents’ distress. A key mechanism, reciprocal regulation, was identified, whereby bereaved parents mirrored the emotional availability or avoidance of their potential support providers. The findings articulate the complexities of social support done well by affirming the importance of attunement.

**Discussion:**

This study offers an expanded understanding of grief as a relationally co-regulated process and calls for improved grief literacy and societal support.

## Introduction

The death of a child, often sudden and typically off-time, is widely regarded as one of the most devastating forms of bereavement (1–4). It poses enduring challenges for adaptation and places parents at heightened risk of adverse biopsychosocial outcomes (5, 6). Between 10 and 25% of bereaved parents experience significant emotional dysregulation (7–10), with prolonged grief disorder (PGD) affecting up to 30%, almost 10 times the prevalence in the general population (11). Common reactions include anxiety, depression, guilt, anger, hopelessness, maladaptive health behaviours (12–14), and diminished workplace participation (15). These persistent stressors demand robust coping strategies.

As one of life’s most profound stressors (16, 17), bereavement requires effective coping strategies to facilitate adaptation. Coping, defined as the cognitive and behavioural efforts to manage stress (18), is conceptualised in Lazarus and Folkman’s transactional model (19) as an oscillation between problem-focused and emotion-focused strategies. Stroebe and Schut’s Dual Process Model (DPM; 20) applied this oscillation to grief, framing adjustment as movement between loss-oriented and restoration-oriented processes. More recently, Guldin and Leget’s Integrated Process Model (IPM; 21) presents grief as an ongoing navigation of tensions across physical, emotional, cognitive, social, and spiritual dimensions. Together, these frameworks highlight that adaptation is not linear but involves ongoing negotiation of intrapersonal and interpersonal demands, shaped by social and cultural contexts.

Among the most critical resources in this process is informal social support. Defined by Cobb (22) as the perception of being loved, valued, and part of a mutual network, informal social support is widely recognised as a buffer against psychological distress (23–30). Support from family, friends, colleagues, and community members (31–33) can buffer distress following bereavement generally (34, 35) and child loss specifically (36, 37). Yet, inadequate attempts at support can also intensify distress (38, 39). Around one-third of bereaved individuals report harm from unhelpful or inadequate support (40), with avoidance, platitudes, or lack of empathy, compounded by providers’ own distress (41–44), often leaving parents feeling more isolated (41, 42). The mismatch between received support (what is offered) and perceived support (its adequacy) is particularly consequential (45–47).

From a social constructionist perspective (48), grief is shaped by cultural norms that dictate how it should be expressed, its duration, and what constitutes ‘healthy’ adaptation. In Western societies, these norms are informed by grief denial (49) and death denial (50). Grief denial reflects discomfort with grief’s intensity, leading to the suppression and marginalisation of grieving individuals (49). Death denial, rooted in Rank (51) and Becker (52), describes unconscious defences against death anxiety, which is linked to existential distress and compulsive behaviours (53, 54). Death anxiety (55, 56), originating in survival-based neural systems (57), is managed culturally through practices that sanitise death and constrain mourning, pressuring bereaved individuals to resume normative roles quickly (58–61). Meanwhile, support providers are expected to care for others while grappling with their own discomfort and fear (45, 62).

Terror Management Theory (TMT; 63, 64) provides a useful lens for understanding these dynamics. It proposes that both bereaved individuals and supporters may conform to cultural norms to reduce existential anxiety. Although conformity may offer temporary relief, it can reinforce rigid expectations that hinder authentic expression and compassionate support. Over-adherence often results in avoidance, strained communication, and emotional distancing; dynamics that ultimately undermine the support process (65). The Interaction Model of Informal Social Support following Bereavement (IM-ISSB; 66) integrates these perspectives, framing support as reciprocal interactions between bereaved individuals and their networks. Helpful interactions strengthen bereaved and network relationships, while unhelpful ones weaken them. However, little is known about how these moment-to-moment exchanges unfold for bereaved parents and their networks, and how they are shaped by broader sociocultural scripts.

### The present study

Although bereaved parents’ support needs are well documented (36, 37), few studies have examined the interactional mechanisms that distinguish helpful from harmful support or explored their embedding in cultural norms. Even fewer apply frameworks such as the IM-ISSB (66), Relational Regulation Theory (RRT; 29), or TMT (63, 64) to interpret these dynamics. Qualitative research is particularly scarce, despite its strength in capturing the lived, moment-to-moment experiences central to understanding support processes in social contexts.

Recent discourse has reframed bereavement support as a community responsibility extending beyond professional care (67). Yet meaningful support cannot be assumed (68). Without adequate skills and confidence, well-intentioned efforts may falter or cause harm (40, 43). Cultivating compassionate communities, therefore, requires transforming the social contexts of grieving and fostering grief literacy, which is a multidimensional skillset encompassing emotional, relational, and cultural competencies (68, 69). This study responds to these gaps by exploring how bereaved parents experience and interpret support interactions following the death of a child. Specifically, it examines parents’ lived experiences of grief, their perceptions of supporters’ responses, and the interactional and cultural mechanisms that facilitate or hinder adaptation.

## Method

This study adhered to the Standards for Reporting Qualitative Research (SRQR; 70) and was cross-referenced against the Consolidated Criteria for Reporting Qualitative Research (COREQ; 71) to ensure comprehensive reporting of study design, researcher reflexivity, context, data collection, analysis, and trustworthiness. The study was conducted from a social constructionist perspective (48) within a constructivist–interpretivist paradigm (72, 73). This paradigm assumes that bereavement experiences are co-constructed through social interaction, embedded within cultural, relational, and contextual frames, and best understood through participants’ narratives interpreted alongside the researchers’ reflexive engagement (73). This lens acknowledges grief as both intrapsychic and interpersonal, situated in sociocultural contexts where meaning, identity, and expectations are negotiated over time. Researcher positionality and reflexivity are therefore viewed as integral to knowledge production rather than as sources of contamination (74).

A qualitative design was selected to capture the lived, interactional nature of bereaved parents’ experiences. Reflexive thematic analysis (75, 76) was chosen for its flexibility and capacity to attend to both semantic and latent meanings, while recognising the researcher’s active role in meaning-making. This approach was particularly suited to the aim of examining interactional and relational processes, as it allows exploration of explicit accounts and deeper interpretive layers.

Four theoretical frameworks provided interpretive scaffolding across the study. The DPM (20) sensitised analysis to oscillation between loss- and restoration-oriented coping. The IPM (21) directed attention to existential tensions such as meaning–meaninglessness and hope–despair. The IM-ISSB (66) highlighted the role of sociocultural grief norms in shaping exchanges of support. RRT (27) emphasised relational regulation and emotional synchrony in interactions. Collectively, these frameworks informed interview guide development, sensitised coding, and supported interpretation by situating moment-to-moment interactions within oscillatory, existential, relational, and cultural processes.

### Researcher characteristics and reflexivity

The first author (JT), a registered psychologist and PhD candidate with extensive experience in grief counselling and bereavement research, conducted all interviews. She identifies as female and was engaged in both academic and clinical practice at the time of data collection. This background facilitated empathic attunement and rapport-building but also carried risks of interpretive influence. To mitigate these risks, reflexivity was embedded throughout the study (70, 71–77). The first author maintained a reflexive journal from study design through analysis, documenting assumptions, evolving interpretations, emotional responses, and potential influences of prior professional knowledge.

Consultation with co-authors provided opportunities to interrogate assumptions, challenge preliminary interpretations, and ensure coding decisions were grounded in participants’ accounts. No prior relationships existed with participants before recruitment. Interviews were conducted as a dedicated research activity, separate from any clinical role, to minimise power differentials and avoid role confusion. Researcher stance was one of interpretive partnership, positioning participants as experts in their own experiences while acknowledging the co-construction of meaning between interviewer and interviewee (74, 75). Rapport was supported through pre-interview check-ins, participant-led choices of timing and modality, and flexibility for pauses or breaks. Emotional safeguards included reminding participants of their right to stop or skip questions, provision of bereavement resource lists at pre-interview and interview conclusion, intentional closing check-ins (“How are you feeling?”; “Do you have support available right now?”), and next-day follow-up where distress was evident. These measures prioritised wellbeing while enhancing integrity and trustworthiness (72, 73).

### Sampling and participants

The study was conducted in Australia (Nov 2024–Feb 2025) within a predominantly White, English-speaking cultural context where informal networks are central to bereavement support. Purposive sampling recruited bereaved parents via bereavement organisations and social media, supplemented by snowball sampling to reach parents outside formal networks. Initial participants were approached by bereavement organisation facilitators acting as gatekeepers. Maximum variation was sought across parental gender, cause of death, age at bereavement, and time since loss to capture a breadth of perspectives.

Eligibility required participants to be aged 18 years or older and to have experienced the death of a child at any time. Sixteen parents participated (13 women, 3 men), all identifying as White Australian (15 in Western Australia, 1 in Victoria). Ages ranged from 35–44 years (*n* = 3), 45–54 (*n* = 3), 55–64 (*n* = 3), to 65 + years (*n* = 7). Causes of death included illness, accident, drug overdose, suicide, sudden death, and stillbirth. Time since the child’s death ranged from 4 months to 38 years (median = 8 years). Although purposive sampling captured a wide range of bereavement experiences, the sample was self-selecting and predominantly mothers, which may limit transferability to fathers and non-binary parents. The sample was also culturally homogenous, reflecting structural barriers to research engagement for culturally and linguistically diverse groups (78). Findings should therefore be interpreted within this Western sociocultural context (79, 80). Sample size was determined pragmatically, guided by thematic sufficiency (77) and Malterud et al.’s (81) principle of ‘information power’ rather than data saturation.

### Ethical considerations

Approval was obtained from the Curtin University Human Research Ethics Committee (HRE2024-0582). Participation was voluntary, with informed consent secured electronically via Qualtrics. Participants were reminded of their right to withdraw at any stage without consequence. Given the sensitivity of the topic, all participants received pre- and post-interview resources for psychological support. No financial incentives were offered to minimise perceived coercion.

### Data collection

Semi-structured interviews were conducted between November 2024 and February 2025 by the first author. Participants chose their preferred timing and modality: eight via Microsoft Teams, seven via telephone, and one in person. Remote options improved accessibility for bereaved parents in metropolitan, regional, and rural locations, reducing barriers to participation. Although video and telephone formats may have influenced rapport, participants reported comfort and openness in sharing through these media. Interviews (60 to 120 min) explored bereavement experiences, perceptions of support, coping, and relational changes.

Initially developed from bereavement literature (62, 82, 83) and theoretical frameworks (20, 21, 27, 66), the interview guide was refined iteratively to allow emerging ideas to shape the inquiry. Doing so is aligned with our epistemology and reflexive thematic analysis. Questions explored participants’ bereavement experiences, perceptions of informal support, coping mechanisms, and relational changes. The guide combined open-ended core questions with prompts to elicit concrete examples and reflective meaning-making. These covered experiences of child loss (e.g., “Can you start by telling me something about your experience since the death of your child?”), support perceptions (e.g., “What form of support/help have you received from friends, family and others?”), coping mechanisms (e.g., “Have you experienced any growth and/or changes since you lost your child?”), and relational changes (e.g., “Have any relationships been lost during your bereavement?”). The format remained flexible to support participant-led narratives, thereby enhancing emotional safety and generating rich, contextualised accounts. Potential power dynamics were mitigated by positioning participants as experts in their experiences and adopting a collaborative, interpretive stance. Interviews were audio-recorded (Microsoft Teams or Otter.ai), transcribed verbatim using Otter.ai, checked for accuracy, anonymised, and securely stored in accordance with university protocols.

### Data analysis

Data were analysed using Braun and Clarke’s (75–77) reflexive thematic analysis, situated within a constructivist–interpretivist paradigm (70–71). This approach acknowledges themes as products of co-construction between researcher and participant, shaped by interpretation and reflexivity. Analysis followed six iterative phases: familiarisation through repeated transcript reading; inductive line-by-line coding; clustering codes into conceptual groupings; reviewing candidate themes for coherence; defining and naming themes to capture analytic essence; and producing the final report with illustrative quotes balancing interpretation with participants’ voices.

Three authors contributed to analysis. The first author coded all transcripts, and two co-authors independently reviewed the developing codes to refine categories, check consistency, and challenge assumptions. Differences in interpretations were resolved through iterative discussion and consensus. NVivo 14 was used for data organisation, consistent with reflexive thematic analysis principles (75–77). Themes were generated inductively, though interpretation was sensitised by grief frameworks (e.g., 20, 21, 27, 66). Attention was given to emotional tone, metaphors, and contradictions, with deviant cases actively examined to refine thematic boundaries. Theme refinement involved comparison across transcripts until coherence and distinctiveness were achieved. The development of themes followed an iterative and reflexive process consistent with reflexive thematic analysis. Early interviews highlighted a broad spectrum of positive and negative support experiences, which initially clustered together under general “helpful” and “unhelpful” categories. As interviews progressed, participants elaborated on the nuanced qualities that distinguished attuned support (e.g., presence, validation, and permission to grieve) from misattuned or harmful support (e.g., avoidance, judgement, or platitudes).

**Figure 1:**
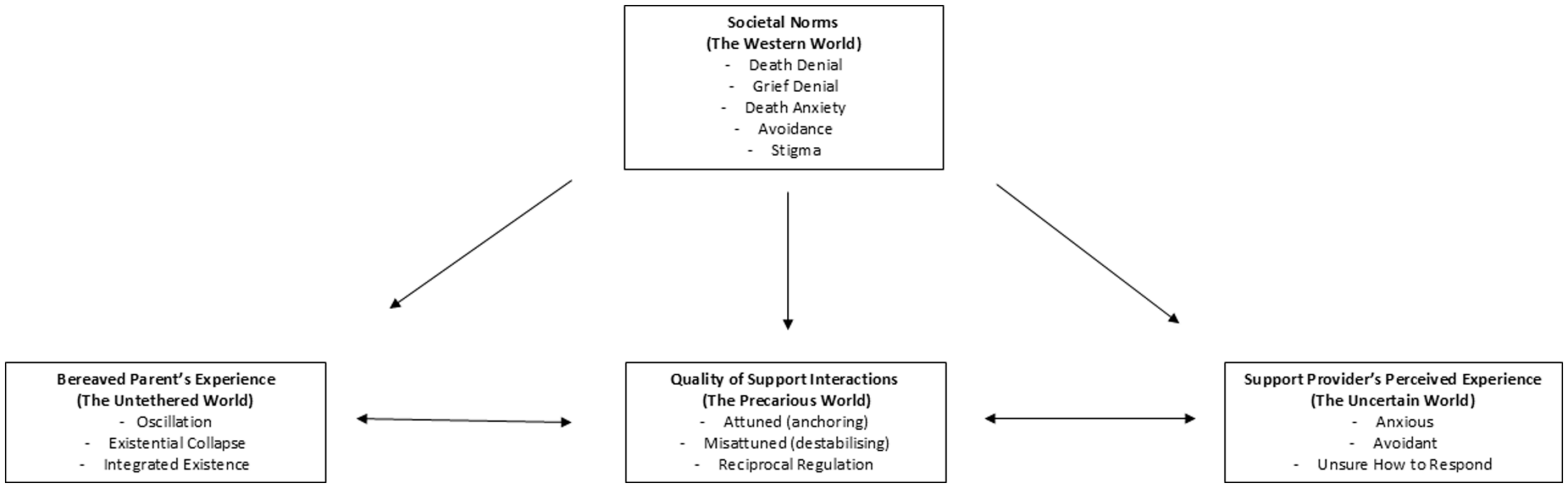
Thematic Map of Themes and Subthemes.

These emergent distinctions informed the refinement of the interview guide, with later interviews probing more specifically into how these differences were experienced and negotiated in everyday interactions. This iterative approach allowed the analysis to move beyond descriptive categorisation toward a deeper understanding of the reciprocal processes that shaped bereaved parents’ experiences of support. Thus, themes became valuable not simply through frequency but through their resonance, explanatory power, and recurrence across participants, refined progressively through cycles of coding, memoing, consultation among the team, and participant feedback. The final thematic map has four overarching themes and associated subthemes (Figure 1). An audit trail of memos, coding logs, and thematic maps documented analytic decisions (see Supplementary materials).

### Rigour and trustworthiness

Rigour was supported through strategies consistent with SRQR (70) and COREQ (71) guidelines, with compliance tables provided in the Supplementary materials. Trustworthiness was further addressed using Lincoln and Guba’s (73) criteria. Credibility was enhanced through prolonged engagement with the data, reflexive journaling, triangulation across interviews, field notes, and analytic memos, and member checking of preliminary thematic summaries. Seven participants provided feedback, ranging from affirmation of resonance (e.g., Ethan: “That certainly resonates”) to constructive critique (e.g., Mary: “Some of your statements resonated with me and some others did not…”). This process was treated not as verification but as a reflexive exchange that enriched interpretation; for example, Ruby’s reflection that “sometimes it feels like we live in the shadows of the grief… because it’s easier for those around us” informed the theme on societal discomfort and avoidance. Olivia likewise highlighted shared resonance: “It’s a comfort to know that it’s just not myself who feels the way I do….”

Dependability was supported through detailed documentation of analytic decisions, including reflexive journals, coding logs, and thematic maps. Confirmability was enhanced through reflexive practice and consultation with co-authors, which challenged assumptions and explored alternative interpretations (70, 71). Transferability was promoted by providing rich description of participant characteristics, contexts, and verbatim quotations to enable readers to assess relevance to other settings (84). Transparency was maintained through detailed documentation of coding iterations, thematic maps, and decision trails. Member checking occurred with preliminary thematic summaries, with participants’ feedback significantly informing refinement and contextual sensitivity. Finally, the quality of thematic analysis was benchmarked against Braun and Clarke’s (85) 15-point checklist (see Supplementary materials). Pseudonyms were used throughout to protect identities.

## Findings

The findings are organised into four interrelated themes: *Societal Norms (The Western World), Bereaved Parents’ Experiences (The Untethered World), Potential Support Providers’ Perceived Experience (The Uncertain World),* and *Quality of Interactions (The Precarious World)*. Member checking confirmed the resonance and validity of this conceptualisation. Olivia wrote, “I think the report absolutely hits the nail right on the head… It’s a comfort to know that it’s not just myself who feels the way I do, which is beautifully summed up in the report, to the exact letter.” Ethan similarly affirmed the framing, noting, “I love the way you have described the experiences as: Untethered, Uncertain, Precarious. That certainly resonates.” Mary also highlighted both the challenge and value of thematic condensation: “You have to be commended for finding four common themes… some of your statements resonated with me and some others did not…” These comments affirmed the thematic core while recognising the inherent heterogeneity of individual grief experiences.

### Societal norms (the Western world)

Parents consistently described navigating a cultural context characterised by discomfort, avoidance, and a demand for restraint. Their grief often triggered the silence and withdrawal of others, revealing Western norms that prioritise stoicism and productivity over vulnerability. Ethan shared, “It’s that silence that almost feels suffocating, because no one knows how to respond… It’s totally their anxiety.” Zara echoed this sentiment: “I think society sticks their head in the sand… It’s just too ugly a topic.” This discomfort left many feeling emotionally abandoned. Ruby reflected, “People do not cope with grief… so they pretend it does not exist,” while Naomi observed, “They change the subject, or they keep quiet.” Stigma, especially around deaths involving suicide or drugs, further isolated parents. Lydia admitted, “I was worried about the opinions of the community,” and Isla noted, “If your child dies by suicide, there’s a fear of getting close to people like us… that it might be contagious.” Liam attributed this avoidance to deeper fears: “They do not want to get the reality because they do not want to think about the possibility of this happening in their own family.”

With regard to societal expectations, Liam suggested, “Impermanence and suffering contrast so starkly with societal norms… Our Western idea is to accumulate, to succeed.” As such, many parents faced pressure to resume normalcy. Zara said, “As soon as Paul’s funeral was done. I went back to work. I’m not getting the space to heal.” Ruby observed, “They give you about a couple of weeks,” and Liam summarised societal expectations: “Be sad for a few months… but really, after a year, you should be over it.” Emotional expression was often met with discomfort. Mary said, “Australians generally are not comfortable if you cry,” and Chloe added, “It’s ‘you’ll be all right, stop that’… just because the other person’s uncomfortable”.

These accounts were reinforced during member checking, where participants described how societal silence and avoidance reverberated through their everyday lives. Ruby concluded, “Sometimes it feels like we live in the shadows of the grief, not because we want to, but because it’s easier for those around us who feel uncomfortable with our grief.” Olivia echoed this resonance, explaining, “It’s a comfort to know that it’s just not myself who feels the way I do…” Together, these cultural scripts shaped how support was offered, withheld, or constrained, intensifying the precarity of the bereaved parent’s experience.

### Bereaved parents’ experiences (the untethered world)

Bereaved parents consistently described their experience as an oscillation between two states: *Existential Collapse* and *Integrated Existence*. Existential Collapse represented profound disintegration across biological, psychological, and social domains. Parents described feeling emotionally paralysed, physically depleted, and existentially disoriented, as though their sense of self, purpose, and identity had been shattered. These moments often involved acute surges of longing, intrusive imagery, and a felt sense of the child’s absence that was so overwhelming it eclipsed daily functioning and the ability to connect with others. In contrast, Integrated Existence captured moments of coherence, where parents were able to sustain an enduring bond with their deceased child while simultaneously re-engaging with life, whether through relationships, work, spiritual practice, or creative expression. This state allowed them to carry the loss in a way that fostered meaning-making, connection, and adaptive functioning. The dynamic and constant movement between the two states was often unpredictable and shaped by how viscerally present or absent their child felt in a given moment.

The depth of Existential Collapse was particularly evident in parents’ accounts of the immediate aftermath of their child’s death, which they experienced as a devastating rupture. Isla captured the cognitive and physical toll: “Deep grief is the inability to think straight,” she said, adding, “I got sicker and sicker… you seem to catch every bug that goes past.” Sophie described public spaces as triggering: “I have most of my panic attacks in Woolies [a supermarket] or Kmart,” and Naomi added, “Everything distresses me. I just walk in the shopping centre, and sometimes I’ve just got to run out.” The enormity of their loss led to overwhelming desolation. Zara reflected, “There’s an awful hole in my life,” and Olivia described it simply as “hell.” Guilt and blame compounded distress. Ethan shared, “It feels like I could not protect him,” and Liam noted, “Blaming and anger gave me a strong sense of connection, so I tended to hang on to them.” Naomi added, “The guilt goes around and around… but she’s dead… there’s nothing I can do.” Over time, grief became nihilistic. Eliza noted, “A lot of things do not matter anymore,” and Mary stated, “Nothing can make up for it… I simply do not care.” She continued, “If I could give every cent that I had… and have Rosie back, I would do it”.

Beyond physical and psychological pain, many described a sense of disorientation. Lydia likened her grief to a phantom limb: “It’s like when someone has an amputee… they still have that connection.” Similarly, Naomi described, “It feels like a piece of my heart has been cut away and I cannot get it.” Some parents described wishing for death or reunion with their child. Freya said, “My hope was death,” and Lydia admitted, “I just want to be with my son. I just do not want to be here anymore.” Similarly, Isla recounted, “You’re wondering how your child is doing… what’s the use of living if they have died?” She summarised this shattered existence as, “We grow up, we have children… and then they die. That’s not right. It turns your order around.” Olivia captured such devastation in her statement, “I’ve had my dark night of the soul.” Time did little to soften this experience. Olivia explained, “People think it gets easier. It does not—it gets worse,” and Liam asserted, “The truth is grief goes on forever.” Social alienation further intensified the psychological collapse. Isla described, “You’re feeling like a stranger in a crowd. normal does not apply anymore,” and Mary exclaimed, “Do not they know the world has changed?” Collectively, these narratives reveal that child loss is a sustained existential crisis that reshapes one’s sense of self, others, and the world. Follow-up reflections during member checking further confirmed this experiential collapse. Freya, despite progress through EMDR therapy, admitted that “all the negative experiences in your summary resonated… unfortunately”.

Despite enduring grief, many parents described ways they integrated grief. Ethan explained, “I made a conscious decision when Kyan passed that I would find grace and beauty in life.” Ilsa described grief as multidimensional: “We have to address the physical, emotional, and spiritual aspects of ourselves, and redefine all three,” and Mary reflected on the transformation process, “You become a deeper, more compassionate, and kind person.” Alongside processing pain, participants described the necessity of continuing daily life, as Ruby noted, “You do not get over it, but you do get on with it”.

Central to this process was maintaining a bond with the deceased child. Jasmine described “doing things that are keeping him alive,” and Liam reframed grief as love: “It’s about love when you flip it around.” Symbolic rituals helped maintain this connection. Freya shared, “We write messages for her, and we send them up with some forget-me-not seeds,” and Ethan emphasised remembrance: “I feel I want to talk about him. I do not want him to be forgotten.” Relationships with their child’s friends also offered a continued connection. Lydia said, “I have a really good relationship with my son’s friends, so I get to see myself grow a bit with them.” Some intimate partnerships strengthened as reflected by Amelia, “The marriage we have is the saving grace… we are just there for each other.” Additionally, peer support was profoundly validating. Jasmine shared, “You hug someone that’s bereaved, and you can just tell by the hug that they get it.” Conversely, many recalibrated their social worlds. Ruby noted, “I have lost a huge circle of friends”.

Grief was described as a non-linear process characterised by oscillation between the states of coherence and collapse. Lydia noted, “You actually do not know how you are feeling… and that feeling can switch from 1 sec to the next,” and Jasmine echoed, “It’s different every day with me.” Social misunderstanding compounded this fluctuation. Jasmine shared, “If you happen to be happy 1 day… then you are down the next, they ask what’s wrong with you.” Meanwhile, grief often resurfaced unexpectedly. Liam said, “It changes and morphs… then may come back to bite you even 10 years later,” and Ruby added, “You’ll smell a smell, and it can take you right back to that moment”.

Emotional expression varied widely. Sophie noted, “Some days I can talk about Benjamin and not cry at all, and some days I cannot even mention his name.” Olivia explained, “I go backwards and forwards in my grief… I might get a little bit sociable, and then I might retreat.” Metaphors captured this shifting terrain. Amelia said, “The waves hit you… they crash into you at the same intense strength they did right when it happened,” and Jasmine described it as “a rollercoaster.” Despite the pain, moments of gratitude and love coexisted with loss. Zara reflected, “I try to connect to the gratitude and the joy of him… sometimes it’s quite easy… and other times it’s really not.” Ultimately, these fluctuations between the existential presence and physical absence of the child shaped whether parents felt grounded in integrated existence or pulled toward existential collapse.

Alongside oscillation between existential collapse and integrated existence, spirituality emerged as a vital regulating tool. Parents described spiritual encounters and practices as means of restoring coherence when faced with overwhelming grief. Lydia reflected, “I cannot stress enough about the spirituality side of things, because it’s not in the textbooks.” For her, spirituality provided a framework for integration that was not available in formal supports. Several parents described continuing bonds with their child through spiritual connection, dreams, or sensing their presence. Others found solace in legacy-building, such as community rituals or symbolic acts. Legacy-building offered meaning and purpose. Amelia shared, “People love that we do so much in Samuel’s name… fundraising and tree planting,” and Olivia described advocacy as “my life work now.” Spirituality was described as the most transformative element. Naomi recalled, “I could feel her lying next to me,” and Amelia summarised, “I’ve never really been particularly spiritual… but now I believe in a greater universe and signs, and that’s what gets me through my days… Samuel sends me signs… and comes to me in dreams… It’s become a new way of living.” These connections helped bridge the gap between enduring existential presence and physical absence. Ruby described this transformation as “It absolutely is my defining moment, because it is pre and it’s post.” Ultimately, integration was ongoing, shaped by enduring love, spiritual connection, and altered world views. These spiritual experiences enabled parents to remain tethered to their child and find moments of coherence amidst collapse, even when external support faltered.

Lydia, in her member-checking feedback, observed that “most, if not all, bereaved parents have experienced some form of spiritual encounter or connection with their child.” She also highlighted the diversity of responses to continuing bonds, noting that although legacy building is “very important to some, others just want to forget and move on.” At the same time, she highlighted that “commonly parents have contemplated suicide themselves.” Eliza highlighted the enduring gap left by child loss, contrasting it with other types of bereavement: “When a child is lost… there’s not the same opportunity to fill the gap that’s been lost.” Taken together, both the original interviews and the member-checking process underscored that child loss constitutes a sustained existential crisis, punctuated only intermittently by moments of integration anchored in enduring love, spirituality, and meaning-making.

### Potential support providers’ perceived experience (the uncertain world)

Following the death of a child, bereaved parents became acutely sensitive to how others internally experienced and responded to their grief. Here, parents turned their gaze outward, speculating on the inner states of those around them, whether they seemed grounded, open and attuned to the parent’s needs, or uncomfortable, avoidant, and misattuned to the parents’ needs. These perceptions shaped how parents navigated their social worlds, informed whom they trusted, and influenced the boundaries they set around emotional exposure.

Some parents described supporters whose inner qualities allowed them to remain steady in the face of profound sorrow. These individuals were perceived as emotionally grounded, authentic, and spiritually open. Lydia appreciated her friend’s capacity for honesty and encouragement: “She does not sugar coat things, you know? She encourages me and has helped me, especially spiritually.” Freya interpreted her supporter’s simple presence as the ability to tolerate grief without fear: “She just held me and I cried, and she just said, you know, just let it go.” For Liam, silence itself was evidence of inner composure: “The bereaved person needs silence… to just be sitting there… and maybe bring a bar of chocolate.” Zara emphasised the rarity of genuine compassion, explaining: “To be in the company of someone who absolutely gets it is a gift. Really. It’s rare.” In these accounts, parents attributed helpful support not only to external behaviours, but to the internal steadiness, honesty, or compassion of the provider. This perceived inner capacity created emotional safety, validating grief without judgement or pressure to “move on”.

Conversely, many parents described interactions that suggested others were unable to emotionally tolerate their grief. These misattuned responses were interpreted as discomfort, avoidance, or self-centring, which intensified parents’ feelings of abandonment. Jasmine reflected on her sister’s silence: “[She] will not mention Jason’s name, and she does not mention Robert at all. I think she does not know what to say.” Zara noted the defensiveness of others: “People do not know what to say, and they are also glad it’s not them… and I do kind of get that.” Parents often attributed avoidance to supporters’ inability to cope, with Lydia observing, “People will cross the street so they do not have to talk to you.” Eliza interpreted silence as self-protective: “People do not bring him up… I think it’s for them. I think it’s to protect them”.

Other examples revealed supporters becoming overwhelmed by their *own* grief, leaving parents feeling displaced. Sophie recalled, “My sister-in-law was a bloody mess… acting like it was her child who had died… she’d message me with all her grief problems.” Ethan acknowledged the limits of compassion without lived experience: “I do not expect parents that have not lost a child to provide support… but I get it.” Sophie added, “If it had not happened to me… I do not know if I would have been the most supportive person.” Some interactions went further, where parents perceived supporters’ internal states as judgemental or blaming. Lydia confided, “He does not love me anymore if he can think that I was the cause of losing our son.” Freya felt silently judged: “I felt like everyone was thinking it was my fault.” Such misattuned inner positions were profoundly wounding, compounding guilt and shame, and reinforcing withdrawal.

Ultimately, parents interpreted platitudes, silence, redirection, or judgement as support provider discomfort and avoidance. These interactions challenged the parents’ sense of relational safety and highlighted the difficulty for support providers to effectively navigate the support exchange process. Lydia’s member checking feedback summarised the emotional toll of this pattern: “Missattuned support [elicits] feelings of abandonment and invalidation… This I am experiencing”.

These perceptions of supporters’ internal states, whether marked by openness, avoidance, or emotional paralysis, did not remain abstract impressions. Rather, they became visible and consequential in the lived dynamics of interaction. In other words, the ways supporters managed their own discomfort or steadiness directly shaped the quality of bereavement support interactions. This transition from internal perception to enacted relational experience is explored in the following theme, the Precarious World, where the fragility of social interactions is revealed through moments of attunement that stabilised parents, or misattunement that deepened their sense of isolation.

### Quality of interactions (the precarious world)

The fourth theme explores how the quality of social interactions shaped bereaved parents’ lived experience of support. Whereas the Potential Support Providers Perceived Experience theme focused on how parents perceived the inner emotional capacities of potential supporters, the Quality of Interactions theme describes how these internal states were enacted in practice and co-regulated in the relational space. Interactions were precarious because they could tip parents toward stability or collapse depending on whether they were experienced as attuned or misattuned.

When supporters’ internal steadiness translated into behaviours of openness, honesty, or gentle presence, interactions were described as profoundly anchoring. Lydia captured this sense of attuned synchrony, “Just that connection without even any words.” Ethan described the power of minimal but embodied presence, “She just put her hand on my arm. And that’s all it took.” Letting parents lead the conversation was key. Lydia explained, “You have to let them release what they want to say,” and Sophie described helpful conversations as characterised by “ebbs and flows.” Ruby valued the freedom to “feel what you need to feel,” and Isla reflected, “My grief was being validated.” Speaking the child’s name was deeply meaningful. Amelia shared, “It brings us so much joy to hear someone say Samuel’s name.” Naomi added, “It does not hurt if you talk about Julie.” Isla remarked, “If you do not talk about Nathan, it’s as if he was never here”.

Symbolic gestures also offered comfort. Ethan shared, “I carried that poem she gave me for so long,” and Amelia appreciated the consistency of a friend: “Sophie has sent me an emoji every single day.” Meanwhile, witnessing other bereaved parents’ adaptation fostered hope. Isla noted, “If they can do it, so can I.” Trust was the foundation of helpful support. Zara expressed, “You just want a safe space… to catch you when you fall.” Crucially, these were not abstract perceptions of supporters’ capacities, but lived moments where grief was shared, mirrored, and held.

Interactions also carried risk. When perceived discomfort in the supporter manifested outwardly as avoidance, platitudes, or judgement, parents described feeling destabilised. Ruby pleaded, “Why are you avoiding me? I just need a hug.” Emotional incompetence compounded the issue. Lydia described awkward probing: “Some people poke the bear… they start shooting questions at you,” which Liam called “a lack of emotional skills or empathy.” Amelia rejected praise framed as strength: “We do not get a choice to be brave.” Passive offers were also frustrating. Chloe questioned, “They say ‘ring me if you need me.’ Why do I have to reach out to you?” Isla added, “We’re the ones that are hurting”.

Judgement and blame were especially damaging. Lydia worried about stigma. “It was a result of taking drugs… are they going to presume he was a drug addict?” Freya recalled, “My brother said… [if you had done something different] maybe she would have been fixed up early.” Moreover, dismissive responses caused distress, with Isla sharing a colleague’s dismissal: “I do not believe in grieving… I just get on with it.” Mismatched grieving styles also caused friction. Mary explained, “He wanted to talk, but I wanted to be with my own grief,” and Freya observed, “We’re on totally different wavelengths.” During member checking, Eliza remarked, “I know also that loss of a child can cause a marriage to break down. I can understand that from my own experience, as I know I have at times withdrawn into myself a lot, and some partners who grieve differently or at a different rate may find this difficult.” Furthermore, disempowerment occurred when grieving choices were overridden. Amelia warned potential support providers, “Do not give advice… even if you have lost a child, you still cannot give advice to that person.” These misattuned interactions often led parents to withdraw socially, intensifying their sense of vulnerability.

What made interactions precarious was the process of reciprocal regulation - the mutual shaping of emotional states between parent and supporter. Lydia explained, “If someone’s made you feel safe… you open up more, and they open up more.” Mary reflected, “It is my responsibility to tell them… unless you let them know, they will not understand… so, you cannot just blame them.” Chloe described co-regulation: “She just held me and I cried.” Mutual support among bereaved parents was particularly meaningful. Olivia said, “We support each other… we see each other’s souls.” Yet over time, parents became discerning about who could “hold” their grief. Parents sometimes sensed the supporter’s discomfort and responded by shielding or suppressing their own grief to protect the other from distress. In these moments, parents were not being comforted but rather regulating the emotional equilibrium of the supporter. Other times, withdrawal was enacted to preserve their own stability. As Chloe explained, “I’ve got to pull away… for my own sanity.” Sophie echoed this, “I definitely do not have the capacity to try anymore.” Such patterns illustrate that regulation was not a one-way process; bereaved parents actively co-regulated the relational field by modulating what they expressed or withheld.

Ultimately, helpful support was not defined by solutions, but by emotional synchrony. Relationships that offered presence, trust, and attunement became steadying anchors whereas those marked by avoidance and misattunement often dissolved. Reciprocal regulation was always in play, either stabilising or destabilising, depending on the quality of the interaction. Member checking feedback corroborated this theme, with Lydia remarking on the importance of “Finding the right fit.” She added, “Parents are very protective of who they tell their story to [and can be left] feeling vulnerable and isolated.” Ultimately, as Lydia’s feedback affirms, the quality of interactions hinged on emotional synchrony, not solutions. When interpersonal support was misattuned or absent, parents often turned to spirituality as a fallback form of regulation. For some, spiritual practices functioned as a substitute for the safety and anchoring that supportive relationships might otherwise provide. In her member checking statement, Lydia emphasised the protective value of spirituality: “Spiritually: most if not all bereaved parents have experienced some form of encounter/connection”.

## Discussion

Bereaved parents in this study described grief as an oscillating, unpredictable process marked by shifts between existential collapse and moments of coherence. Their experiences align with contemporary grief models, particularly Stroebe and Schut’s DPM (20), which frames adaptive coping as oscillation between loss- and restoration-oriented processes. However, participants’ descriptions extended beyond functional coping strategies, revealing deeper existential instability, where grief disrupted their sense of self, others, and the world. To better account for this experience, Guldin and Leget’s IPM (21) adds nuance by identifying five existential polarities: meaning vs. meaninglessness, connection vs. isolation, order vs. chaos, control vs. helplessness, and hope vs. despair. The IPM views these tensions as persistent, lived polarities that bereaved individuals must continually navigate. Rather than being tasks to complete, these tensions are recurring experiential movements. Importantly, the IPM foregrounds the non-pathological nature of oscillation, presenting it as the very terrain through which adaptation unfolds (21).

Parents navigated these tensions continuously, often expressing competing needs, such as speaking openly versus protecting themselves, or connecting spiritually while simultaneously confronting despair. These existential fluctuations resonate with Relational Dialectics Theory (86), which conceptualises relationships as ongoing negotiations of opposing needs. Consistent with findings by Toller (87) and Hooghe et al. (88), participants oscillated between openness and emotional withdrawal, calibrating their level of expression based on the perceived safety of their relational context. Supportive responses—those marked by compassion and non-judgement—tended to foster emotional openness in the bereaved parents. In contrast, when interactions were coloured by judgement, discomfort, or misunderstanding, the bereaved parents often withdrew or silenced themselves.

Crucially, this study extends existing knowledge by illuminating *why* potential support may fail to help: it is not merely the presence or absence of support that matters, but how well it aligns with the bereaved person’s fluctuating relational and emotional needs. The dynamic interplay between bereaved parents and their informal support networks revealed that even well-intended support could be unhelpful when it fails to attune to these oscillations. This insight highlights the novel contribution of our study, shedding light on the interpersonal micro-dynamics that underlie failed support interactions and offering a nuanced understanding of how and why relational support interactions may both help and harm in the context of grief.

This relational dissonance was not only interpersonal but deeply existential, echoing broader philosophical-phenomenological accounts that frame grief as a fundamental disruption to one’s experience of the world. These accounts, particularly those of Ratcliffe (89), Brison (90), Humphreys (91), and Attig (92), offer further insight into the altered fabric of the bereaved’s existence. Ratcliffe (89) suggested grief alters the very structure of lived experience, creating a world suffused with a “spectral existence” of the deceased. Participants in this study exhibited this phenomenon, describing feeling the presence of their child as a persistent yet intangible absence, neither fully present nor entirely gone. Meanwhile, Attig (92) defines grief as a process of relearning the world, and participants reported repeated and painful confrontations with a world rendered unfamiliar and devastated by the absence of their child.

Emerging within this existential rupture is our core finding of reciprocal regulation, which is the mutual influence between bereaved parents and support providers. While grief reconfigured participants’ worlds, their social interactions with others either anchored them momentarily within that world or exacerbated their distress. Within a broader sociocultural context marked by grief and death denial, these interactions became emotionally charged exchanges. The concept of relational regulation (27) captures this dynamic, where attunement or misattunement functions as a mechanism of emotional mirroring. Attuned social support, defined by emotionally safe presence, validation, and acknowledgment of the parents’ continuing bond with their child, helped parents remain anchored in moments of coherence and connection. Conversely, misattuned interactions, characterised by avoidance, incompetence, or discomfort, were typically mirrored by the parent, prompting their silence, withdrawal, or disengagement. Some participants perceived such responses as stemming from unacknowledged death anxiety, which posits that grief can provoke defensive reactions in others when mortality salience is high (56).

Although “attunement” is often used descriptively in bereavement research, our findings highlight the need to unpack what it entails in practice. Attunement refers not simply to the provision of support, but to the supporter’s capacity to sense accurately, resonate with, and flexibly respond to the bereaved parent’s fluctuating emotional state. Consistent with Barboza et al. (93) and Barboza and Seedall (94), attunement can be understood as a dynamic process of relational resonance, in which compassion, emotional availability, and responsiveness converge to create a sense of being understood and accompanied. Parents described attunement as occurring when supporters were able to be fully present, validate their grief, and tolerate emotional intensity without avoidance, judgement, or pressure to “move on.” Mechanisms underpinning attunement included affective compassion, experiential resonance (particularly within peer relationships), and behavioural adaptability that allowed the bereaved to oscillate naturally between silence and disclosure, grief and restoration.

Attunement was often difficult for supporters to sustain because bereaved parents’ grief was itself oscillatory and unpredictable. As parents moved between collapse and coherence, their needs for closeness, distance, or silence shifted, sometimes rapidly. Supporters who lacked tolerance for this ambiguity often withdrew or defaulted to platitudes. As Barboza et al. (93) suggested, attunement requires comfort with emotional complexity, ambiguity, and loss of control—qualities not well supported in Western cultures shaped by grief denial and death anxiety. Our findings also suggest that withdrawal can function in two ways: as a self-protective form of regulation (e.g., “I needed to pull away for my sanity”) and as a co-regulatory strategy aimed at protecting others (e.g., masking grief to shield supporters from discomfort). These dual roles highlight the fragility of attunement and explain why it is both deeply valued and often absent.

The significance of reciprocal regulation becomes even more apparent when considered alongside the psychological vulnerabilities commonly experienced by bereaved parents. Many parents described symptoms consistent with diagnostic criteria for PTSD, PGD, and depression (95). Regularly co-occurring with the death of a child (8, 11), these disorders amplify emotional vulnerability and relational sensitivity, rendering the quality of interpersonal interactions especially consequential. Among the parents, symptoms of PTSD, such as hypervigilance, intrusive thoughts, and emotional dysregulation, characteristically increased reactivity to perceived emotional incompetence or relational threat, consistent with prior findings (96, 97). Similarly, the core features of PGD, including persistent yearning, identity disintegration, and existential despair, were frequently intensified by dismissive, invalidating, or judgemental responses, an observation that aligns with established literature (11). Depressive symptoms such as anhedonia, hopelessness, and social withdrawal further constrained parents’ capacity to seek and accept support, limiting opportunities for co-regulation, as observed by Vance et al. (13). In contrast, attuned interactions created spaces of safe containment, allowing parents to remain relationally, physically, and spiritually tethered.

A notable nuance in the findings concerns parents’ descriptions of wishing for death or reunion with their child. Although some accounts were initially coded as suicidal thoughts (e.g., “I just do not want to be here anymore”), closer reflection suggested that many of these were more accurately *death-related fantasies*. These expressions are well-documented in grief literature, especially among bereaved parents, and reflect a longing for reunion or an escape from suffering rather than an intent or plan to enact self-harm (98–100). Distinguishing between suicidality and fantasies of death is clinically important, as the latter are typically an aspect of the yearning that defines grief rather than indicators of psychiatric crisis. This nuance underscores the need for support providers, clinicians, and researchers to interpret parents’ words within the relational and existential context of bereavement, ensuring that expressions of longing are understood and responded to with sensitivity rather than automatically pathologised.

Amid these psychological and relational vulnerabilities, many parents turned to spirituality as a crucial resource for regulation, enabling a continued connection with their child that helped restore coherence in the aftermath of profound loss. Fourteen of the 16 participants described spiritual experiences, such as dreams and signs as vital to sustaining a sense of connection with their child. These experiences are consistent with the continuing bonds (CB) framework (101), which emphasises the symbolic and relational persistence of the deceased in the lives of the bereaved. These spiritually mediated connections resembled *internalised* CB expressions, involving symbolic representations of the deceased maintained through memory, imagination, or felt presence (102). Internalised forms of continuing bonds have been shown to reduce grief intensity and support psychological adjustment, particularly for bereaved parents (103, 104). Spirituality, in this context, functioned both as a regulatory and integrative force, offering a framework for identity reconstruction, existential grounding, and ongoing attachment to the deceased child. While some participants drew on traditional religious narratives, others embraced intuitive, personalised forms of spirituality, consistent with Burke and Neimeyer’s (105) findings on individualised coping.

In contrast, *externalised* CB expressions, such as seeing or hearing the deceased, preserving their possessions unchanged, or engaging in ritualistic behaviours, have been linked to heightened distress and poorer grief outcomes, particularly in early or unresolved grief (104, 106–109). These expressions are marked by efforts to sustain a concrete connection with the deceased, reflect difficulty accepting the loss (106), and are common among parents whose children died suddenly or violently (108). Several participants who reported externalised CBs also described symptoms consistent with PTSD and PGD, reinforcing their associations with negative grief outcomes (107–109).

Overall, participants demonstrated use of both internalised and externalised CBs, which corresponded to differing grief trajectories. Consistent with Field et al. (108), spiritual experiences, particularly those involving transcendent or symbolic connections, were frequently perceived by the parents as comforting and stabilising. As such, spirituality may be understood as a protective regulatory process that supports the transformation of externalised grief into an internalised, adaptive, and enduring bond (110–112).

These relational and spiritual findings converge within the IM-ISSB (66), which conceptualises informal social support attempts as a reciprocal process shaped by individual, relational, and sociocultural factors. According to this model, effective support is marked by openness, sensitivity, and self-efficacy. Participants’ accounts affirmed the IM-ISSB’s core claim that attuned support enables mutual regulation, trust, and reintegration. Conversely, the model also posits that fear, avoidance, or cultural taboos undermine the potential for connection and integration. Congruent with this assertion, most parents described potential support providers as often emotionally paralysed, fearful of saying the wrong thing or overwhelmed by their own discomfort. These responses reflect broad Western cultural scripts that tend to demand rapid adaptation, suppress grief, and reward productivity over presence (49–51).

Such sociocultural dynamics are further illuminated by Terror Management Theory (63, 64), which offers a psychological explanation for the avoidance and discomfort observed in many support providers. It posits that death anxiety elicits defensive reactions such as denial, rationalisation, or retreat into cultural worldviews that promote control, growth, and permanence. When confronted with raw, enduring grief such as the loss of a child, these buffers are threatened, and support providers may instinctively minimise, distract, or withdraw. For bereaved parents, this retreat is often experienced as abandonment or invalidation, further complicating their grief trajectory.

Recognising the influence of these underlying defence mechanisms, it becomes essential to explore how such dynamics manifest across different cultural and demographic groups, and how support providers themselves experience and respond to bereavement-related distress. These future directions emerge within a broader societal context marked by growing interest in compassionate communities (67). Although this framework rightly emphasises the importance of social networks in bereavement support, it risks assuming that individuals and communities already possess the knowledge and emotional resources to provide meaningful and effective social support (68). However, as the present study showed, families and friends are not always well-equipped to meet the complex and evolving needs of the bereaved (40, 41, 68). A paradigm shift is needed that moves beyond rhetorical endorsements of communal care toward meaningful investment in both specialised services and community capacity. This involves expanding the breadth of care so that specialised services are developed and available, while also building the community’s capacity to provide responsive and compassionate support (68). Only by fostering relational attunement and transforming the social contexts within which grieving occurs can the ideals of compassionate communities be achieved.

### Limitations

Despite employing strategies to enhance trustworthiness, including reflexivity, member checking, and an audit trail, several limitations must be acknowledged. The sample consisted primarily of mothers (13 of 16), potentially overrepresenting maternal perspectives. Participants were all based in Australia and recruited through bereavement networks and snowballing, possibly attracting parents more engaged in support-seeking and limiting transferability to those who grieve privately. Perspectives from culturally and linguistically diverse communities, Aboriginal and Torres Strait Islander peoples, and parents at very early or prolonged stages of grief were underrepresented. Data were self-reported through in-depth interviews, aligning with the study’s focus on subjective meaning-making but not capturing observed behaviours (113). Microsoft Teams meetings and telephone calls may also have shaped rapport, though participants reported feeling comfortable.

Findings were co-constructed within a reflexive thematic analysis framework (75, 76), meaning researcher positionality shaped interpretation. Reflexivity, debriefing, and member checking mitigated this. Although member checking enhanced credibility due to the affirming nature of the feedback, only some participants responded, meaning that our interpretations might not reflect all perspectives. Notably, the findings are situated within a Western sociocultural context where grief is marginalised, and stoicism prioritised. Concepts such as continuing bonds or societal avoidance may not translate across cultures, particularly in collectivist contexts with communal grief practices (114). As such, the findings provide contextually rich insights that may be transferable where resonance is recognised (73). Importantly, the sample consisted entirely of White Australian parents. Although recruitment was open to all, this outcome likely reflects wider barriers to participation for minority and Indigenous populations, including stigma, mistrust, and reduced access to bereavement networks (115). Cultural norms strongly shape grieving practices and support processes; for example, communal and ritualised forms of support common in collectivist contexts may foster different dynamics of reciprocal regulation than those observed here in a Western setting (114, 116, 117). Future research should therefore explore how attunement, misattunement, and continuing bonds manifest across in communities where cultural scripts for grieving may create distinct opportunities and challenges for support.

Finally, this study captured only the perspectives of bereaved parents as recipients of informal social support. While this focus offers valuable insight into their lived experiences, informal support exchanges are inherently relational. Future research should aim to examine both perspectives in these dyadic interactions. Investigating both sides of the exchange (i.e., perspectives of bereaved parents and people who aim to support them) would generate a fuller understanding of how attunement or misattunement arise in practice and illuminate the relational dynamics that either facilitate or hinder meaningful support. Such dyadic approaches would also provide stronger foundations for grief literacy initiatives by directly informing training and resources that are responsive to the needs of both providers and recipients.

## Conclusion

This study explored how bereaved parents navigate grief following the death of a child, identifying four intersecting “worlds”: the Western World (Societal Norms), the Untethered World (Bereaved Parents’ Experience), the Uncertain World (Perceived Support Provider Experience), and the Precarious World (Quality of Interactions). Across these domains, the process of reciprocal regulation emerged as central. Attuned, consistent, and non-directive support offered grounding, whereas avoidance, judgement, or insensitivity intensified collapse and isolation. Situated within a Western sociocultural context that often silences grief, many parents sought solace in spiritual or symbolic continuing bonds to remain connected to their child. These findings highlight grief as simultaneously individual, relational, and cultural, shaped by the capacity of others to respond with attuned presence rather than misattuned avoidance. Implications extend to policy and practice. Building community grief literacy, equipping support providers with interactional and communication skills, and strengthening peer networks and professional services can create environments where grief is acknowledged rather than marginalised. By fostering reciprocal, attuned support, communities can mitigate isolation and promote long-term well-being for bereaved parents.

## Data Availability

The raw data supporting the conclusions of this article will be made available by the authors, without undue reservation.

## References

[1] American Psychiatric Association. Diagnostic and statistical manual of mental disorders. 3rd ed. Washington, DC: American Psychiatric Association (1987).

[2] Dyregrov K Nordanger D Dyregrov A. Predictors of psychosocial distress after suicide, SIDS and accidents. Death Stud. (2003) 27:143–65. doi: 10.1080/07481180302892, PMID: 12678058

[3] Meert KL Shear K Newth CJL Harrison R Berger J Zimmerman J. Follow-up study of complicated grief among parents eighteen months after a child’s death in the Pediatric intensive care unit. J Palliat Med. (2011) 14:207–14. doi: 10.1089/jpm.2010.0291, PMID: 21281122 PMC3037801

[4] Snaman JM Kaye EC Torres C Gibson D Baker JN. Parental grief following the death of a child from cancer: the ongoing odyssey. Pediatr Blood Cancer. (2016) 63:1594–602. doi: 10.1002/pbc.26046, PMID: 27187020

[5] Middleton W Raphael B Martinek N Burnett P. A longitudinal study comparing bereavement phenomena in recently bereaved spouses, adult children and parents. Aust N Z J Psychiatry. (1998) 32:235–41. doi: 10.3109/00048679809062734, PMID: 9588303

[6] Aoun SM Breen LJ Howting DA Rumbold B McNamara B Hegney D. Who needs bereavement support? A population based survey of bereavement risk and support need. PLoS One. (2015) 10:10. doi: 10.1371/journal.pone.0121101, PMID: 25811912 PMC4374848

[7] Kaltman S Bonanno GA. Trauma and bereavement: examining the impact of sudden and violent deaths. J Anxiety Disord. (2003) 17:131–47. doi: 10.1016/S0887-6185(02)00184-6, PMID: 12614658

[8] Kristensen P Weisæth L Heir T. Bereavement and mental health after sudden and violent losses: a review. Psychiatry Interpers Biolog Processes. (2012) 75:76–97. doi: 10.1521/psyc.2012.75.1.76, PMID: 22397543

[9] October T Dryden-Palmer K Copnell B Meert KL. Caring for parents after the death of a child. Pediatr Crit Care Med. (2018) 19:S61–8. doi: 10.1097/PCC.0000000000001466, PMID: 30080812 PMC6082144

[10] Wiener L Rosenberg AR Lichtenthal WG Tager J Weaver MS. Personalized and yet standardized: an informed approach to the integration of bereavement care in pediatric oncology settings. Palliat Support Care. (2018) 16:706–11. doi: 10.1017/S1478951517001249, PMID: 29386073 PMC6070438

[11] Shear MK. Complicated grief. N Engl J Med. (2015) 372:153–60. doi: 10.1056/NEJMcp1315618, PMID: 25564898

[12] Rubin S Malkinson R. Parental response to child loss across the life cycle: clinical and research perspectives In: Stroebe M Hansson R Stroebe W Schut H, editors. Handbook of bereavement research: Consequences, coping, and care. Washington, DC: American Psychological Association (2001). 219–40.

[13] Vance JC Najman JM Boyle FM Embelton G Foster WJ Thearle MJ. Alcohol and drug usage in parents soon after stillbirth, neonatal death or SIDS. J Paediatr Child Health. (1994) 30:269–72. doi: 10.1111/j.1440-1754.1994.tb00632.x, PMID: 8074915

[14] Videka-Sherman L. Coping with the death of a child: a study over time. Am J Orthopsychiatry. (1982) 52:688–98. doi: 10.1111/j.1939-0025.1982.tb01458.x, PMID: 7148990

[15] van den Berg GJ Lundborg P Vikström J. The economics of grief. Econ J. (2017) 127:1794–832. doi: 10.1111/ecoj.12399, PMID: 40916555

[16] Holmes TH Rahe RH. The social readjustment rating scale. J Psychosom Res. (1967) 11:213–8. doi: 10.1016/0022-3999(67)90010-4, PMID: 6059863

[17] Stroebe M Schut H Stroebe W. Health outcomes of bereavement. Lancet. (2007) 370:1960–73. doi: 10.1016/S0140-6736(07)61816-9, PMID: 18068517

[18] Folkman S. Revised coping theory and the process of bereavement In: Folkman S, editor. Handbook of Bereavement research: Consequences, coping, and care. Washington, DC: American Psychological Association (2001). 563–84.

[19] Lazarus RS Folkman S. Transactional theory and research on emotions and coping. Eur J Personal. (1987) 1:141–69. doi: 10.1002/per.2410010304, PMID: 40916550

[20] Stroebe M Schut H. The dual process model of coping with bereavement: rationale and description. Death Stud. (1999) 23:197–224. doi: 10.1080/074811899201046, PMID: 10848151

[21] Guldin MB Leget C. The integrated process model of loss and grief – an interprofessional understanding. Death Stud. (2024) 48:738–52. doi: 10.1080/07481187.2023.2272960, PMID: 37883693

[22] Cobb S. Social support as a moderator of life stress. Psychosom Med. (1976) 38:300–14. doi: 10.1097/00006842-197609000-00003, PMID: 981490

[23] Barrera M. Distinctions between social support concepts, measures, and models. Am J Community Psychol. (1986) 14:413–45. doi: 10.1007/BF00922627

[24] Breen LJ. Harnessing social support for bereavement now and beyond the COVID-19 pandemic. Palliat Care Soc Pract. (2021) 15:1–4. doi: 10.1177/2632352420988009, PMID: 34104884 PMC8164552

[25] Cohen S Wills TA. Stress, social support, and the buffering hypothesis. Psychol Bull. (1985) 98:310–57. doi: 10.1037/0033-2909.98.2.310, PMID: 3901065

[26] Hupcey JE. Clarifying the social support theory-research linkage. J Adv Nurs. (1998) 27:1231–41. doi: 10.1046/j.1365-2648.1998.01231.x, PMID: 9663875

[27] Lakey B Orehek E. Relational regulation theory: a new approach to explain the link between perceived social support and mental health. Psychol Rev. (2011) 118:482–95. doi: 10.1037/a0023477, PMID: 21534704

[28] Scott HR Pitman A Kozhuharova P Lloyd-Evans B. A systematic review of studies describing the influence of informal social support on psychological wellbeing in people bereaved by sudden or violent causes of death. BMC Psychiatry. (2020) 20:1–20. doi: 10.1186/s12888-020-02639-4, PMID: 32471407 PMC7257446

[29] Taylor S. Social support: a review In: Friedman HS, editor. The Oxford handbook of Health Psychology. Oxford: Oxford University Press (2020). 189–214.

[30] Wang J Lloyd-Evans B Giacco D Forsyth R Nebo C Mann F. Social isolation in mental health: a conceptual and methodological review. Soc Psychiatry Psychiatr Epidemiol. (2017) 52:1451–61. doi: 10.1007/s00127-017-1446-1, PMID: 29080941 PMC5702385

[31] Aoun SM Breen LJ White I Rumbold B Kellehear A. What sources of bereavement support are perceived helpful by bereaved people and why? Empirical evidence for the compassionate communities approach. Palliat Med. (2018) 32:1378–88. doi: 10.1177/0269216318774995, PMID: 29754514 PMC6088515

[32] Dyregrov A Dyregrov K. Effective grief and bereavement support: The role of family, friends, colleagues, schools and support professionals. London: Jessica Kingsley Publishers (2008).

[33] Wills T. Social support and interpersonal relationships In: Clark MS, editor. Review of personality and social psychology: Prosocial behavior, vol. 12. Newbury Park, CA: Sage (1991). 265–89.

[34] Chen R. Social support as a protective factor against the effect of grief reactions on depression for bereaved single older adults. Death Stud. (2022) 46:756–63. doi: 10.1080/07481187.2020.1774943, PMID: 32496893

[35] Lobb EA Kristjanson LJ Aoun SM Monterosso L Halkett GKB Davies A. Predictors of complicated grief: a systematic review of empirical studies. Death Stud. (2010) 34:673–98. doi: 10.1080/07481187.2010.496686, PMID: 24482845

[36] Schoonover KL Prokop L Lapid MI. Valuable informal bereavement support strategies for bereaved parents of stillborn, young children, and adult children: a scoping review. J Palliat Care. (2022) 37:381–400. doi: 10.1177/08258597211062762, PMID: 35354346

[37] Wang E Hu H He Y Xu Y. Can social support matter? the relationship between social support and mental health among bereaved parents in an only-child society: evidence from China. Health Soc Care Community. (2021) 29:476–86. doi: 10.1111/hsc.13108, PMID: 32701221

[38] Bolger N Amarel D. Effects of social support visibility on adjustment to stress: experimental evidence. J Pers Soc Psychol. (2007) 92:458–75. doi: 10.1037/0022-3514.92.3.458, PMID: 17352603

[39] Bolger N Zuckerman A Kessler RC. Invisible support and adjustment to stress. J Pers Soc Psychol. (2000) 79:953–61. doi: 10.1037/0022-3514.79.6.953, PMID: 11138764

[40] Aoun SM Keegan O Roberts A Breen LJ. The impact of bereavement support on wellbeing: a comparative study between Australia and Ireland. Palliat Care Soc Pract. (2020) 14:132. doi: 10.1177/2632352420935132, PMID: 32783026 PMC7385836

[41] Breen LJ O’Connor M. Family and social networks after bereavement: experiences of support, change and isolation. J Fam Ther. (2011) 33:98–120. doi: 10.1111/j.1467-6427.2010.00495.x

[42] Grindrod A Rumbold B. Healthy end of life project (HELP): a progress report on implementing community guidance on public health palliative care initiatives in Australia. Ann Palliat Med. (2018) 7:S73–83. doi: 10.21037/apm.2018.04.01, PMID: 29764174

[43] Breen LJ Aoun SM Rumbold B McNamara B Howting DA Mancini V. Building community capacity in bereavement support. Am J Hosp Palliat Med. (2017) 34:275–81. doi: 10.1177/1049909115615568, PMID: 26566928

[44] Smith KV Wild J Ehlers A. The masking of mourning: social disconnection after bereavement and its role in psychological distress. Clin Psychol Sci. (2020) 8:464–76. doi: 10.1177/2167702620902748, PMID: 32550046 PMC7252572

[45] Kaplan J Block R Gillard A Putnam M. Inventory of youth adaptation to loss (IYAL): psychometric testing of a new instrument for bereaved youth to assess social support and coping. OMEGA. (2022) 86:503–32. doi: 10.1177/0030222820976299, PMID: 33283630

[46] Lehman DR Ellard JH Wortman CB. Social support for the bereaved: recipients’ and providers’ perspectives on what is helpful. J Consult Clin Psychol. (1986) 54:438–46. doi: 10.1037//0022-006X.54.4.438

[47] Dyregrov K. Experiences of social networks supporting traumatically bereaved. OMEGA. (2006) 52:339–58. doi: 10.2190/CLAA-X2LW-JHQJ-T2DM, PMID: 22612255

[48] Berger PL Luckmann T. The social construction of. Reality: A treatise in. New York: Penguin Books (1966).

[49] Macdonald ME. The denial of grief In: Jacobsen M Petersen A, editors. Exploring Grief. Abingdon: Routledge (2019). 125–39.

[50] Zimmermann C Rodin G. The denial of death thesis: sociological critique and implications for palliative care. Palliat Med. (2004) 18:121–8. doi: 10.1191/0269216304pm858oa, PMID: 15046408

[51] Raphael T. seelenglaube und psychologie. by dr. Otto Rank. (leipzig und Wien: Franz Deuticke). Am J Psychiatry. (1930) 88:400–13. doi: 10.1176/ajp.88.2.400, PMID: 40235603

[52] Becker E. The denial of death. New York: Free Press (1973).

[53] Menzies RE Dar-Nimrod I. Death anxiety and its relationship with obsessive-compulsive disorder. J Abnorm Psychol. (2017) 126:367–77. doi: 10.1037/abn0000263, PMID: 28277734

[54] Menzies RE Sharpe L Dar-Nimrod I. The relationship between death anxiety and severity of mental illnesses. Br J Clin Psychol. (2019) 58:452–67. doi: 10.1111/bjc.12229, PMID: 31318066

[55] Greenberg J Pyszczynski T Solomon S Simon L Breus M. Role of consciousness and accessibility of death-related thoughts in mortality salience effects. J Pers Soc Psychol. (1994) 67:627–37. doi: 10.1037/0022-3514.67.4.627, PMID: 7965609

[56] Pandya A Kathuria T. Death anxiety, religiosity and culture: implications for therapeutic process and future research. Religion. (2021) 12:61. doi: 10.3390/rel12010061

[57] Panksepp J. Affective neuroscience: The foundations of human and animal emotions. Oxford: Oxford University Press (1998).

[58] Gudmundsdottir M Chesla CA. Building a new experience. J Fam Nurs. (2006) 12:143–64. doi: 10.1177/1074840706287275 16621783

[59] Martz E Livneh H. Death anxiety as a predictor of future time orientation among individuals with spinal cord injuries. Disabil Rehabil. (2003) 25:1024–32. doi: 10.1080/09638280310001596469, PMID: 12944157

[60] Umphrey LR Cacciatore J. Coping with the ultimate deprivation: narrative themes in a parental bereavement support group. OMEGA. (2011) 63:141–60. doi: 10.2190/OM.63.2.c, PMID: 21842663

[61] Walsh F. Loss and bereavement in families: a systemic framework for recovery and resilience In: Fiese BH Celano M Deater-Deckard KD Jouriles EN Whisman MA, editors. APA handbook of contemporary family psychology: Foundations, methods, and contemporary issues across the lifespan, vol. 1. Washington, DC: American Psychological Association (2019). 649–63.

[62] Dyregrov K Kristensen P Dyregrov A. A relational perspective on social support between bereaved and their networks after terror: a qualitative study. Glob Qual Nurs Res. (2018) 5:2076. doi: 10.1177/2333393618792076, PMID: 30116765 PMC6088469

[63] Solomon S Greenberg J Pyszczynski T. A terror management theory of social behavior: the psychological functions of self-esteem and cultural worldviews. Adv Exp Soc Psychol. (1991) 24:93–159. doi: 10.1016/S0065-2601(08)60328-7

[64] Solomon S Greenberg J Pyszczynski T. The worm at the core: On the role of death in life. New York: Penguin Random House (2015).

[65] Moreton J Kelly CS Sandstrom GM. Social support from weak ties: insight from the literature on minimal social interactions. Soc Personal Psychol Compass. (2023) 17:12729. doi: 10.1111/spc3.12729

[66] Tognela JA Rudaizky D Robinson KT Mason HM Breen LJ. Informal social support following bereavement: a scoping review of provider and recipient perspectives of helpful and unhelpful interactions. Death Stud. (2025) 2025:1–14. doi: 10.1080/07481187.2025.2454506, PMID: 39876766

[67] Mills J Abel J Kellehear A Noonan K Bollig G Grindod A. The role and contribution of compassionate communities. Lancet. (2024) 404:104–6. doi: 10.1016/S0140-6736(23)02269-9, PMID: 37844589

[68] Breen LJ Kawashima D Joy K Cadell S Roth D Chow A. Grief literacy: a call to action for compassionate communities. Death Stud. (2022) 46:425–33. doi: 10.1080/07481187.2020.1739780, PMID: 32189580

[69] Maccallum F Breen LJ Ivynian S DiGiacomo M Luckett T Lobb EA. Prolonged grief reactions and help-seeking in bereaved adults during the COVID-19 pandemic. J Affect Disord. (2025) 374:467–76. doi: 10.1016/j.jad.2025.01.056, PMID: 39809353

[70] O’Brien BC Harris IB Beckman TJ Reed DA Cook DA. Standards for reporting qualitative research. Acad Med. (2014) 89:1245–51. doi: 10.1097/acm.0000000000000388, PMID: 24979285

[71] Tong A Sainsbury P Craig J. Consolidated criteria for reporting qualitative research (COREQ): a 32-item checklist for interviews and focus groups. Int J Qual Health Care. (2007) 19:349–57. doi: 10.1093/intqhc/mzm042, PMID: 17872937

[72] Schwandt TA. Constructivist, interpretivist approaches to human inquiry In: Denzin NK Lincoln YS, editors. Handbook of qualitative research. Thousand Oaks, CA: SAGE (1994). 118–37.

[73] Lincoln YS Guba EG. Naturalistic inquiry. Thousand Oaks, CA: SAGE (1985).

[74] Levitt HM Motulsky SL Wertz FJ Morrow SL Ponterotto JG. Recommendations for designing and reviewing qualitative research in psychology: promoting methodological integrity. Qual Psychol. (2017) 4:2–22. doi: 10.1037/qup0000082

[75] Braun V Clarke V. Using thematic analysis in psychology. Qual Res Psychol. (2006) 3:77–101. doi: 10.1191/1478088706qp063oa

[76] Braun V Clarke V. Successful qualitative research: A practical guide for beginners. Thousand Oaks, CA: SAGE Publications Ltd. (2013).

[77] Braun V Clarke V. One size fits all? What counts as quality practice in (reflexive) thematic analysis? Qual Res Psychol. (2021) 18:328–52. doi: 10.1080/14780887.2020.1769238

[78] Australian Government. *Reporting on the health of culturally and linguistically diverse populations in Australia: An exploratory paper, Summary-Australian Institute of Health and Welfare*. (2022). Available online at: https://www.aihw.gov.au/reports/cald-australians/reporting-health-cald-populations/summary (Accessed August 19, 2025).

[79] What WT. Death means now: Thinking critically about dying and grieving. Bristol, UK: Policy Press (2018).

[80] Klass D Silverman PR Nickman S. Continuing bonds: New understandings of grief. Abingdon: Routledge (2014).

[81] Malterud K Siersma VD Guassora AD. Sample size in qualitative interview studies. Qual Health Res. (2016) 26:1753–60. doi: 10.1177/1049732315617444, PMID: 26613970

[82] Aoun SM Breen LJ Rumbold B Christian KM Same A Abel J. Matching response to need: What makes social networks fit for providing bereavement support? PLoS One. (2019) 14:14. doi: 10.1371/journal.pone.0213367, PMID: 30845193 PMC6405096

[83] Morrigan B Keesing S Breen LJ. Exploring the social networks of bereaved spouses: phenomenological case studies. OMEGA. (2022) 85:268–84. doi: 10.1177/0030222820944062, PMID: 32698677

[84] Geertz C. Thick description: toward an interpretive theory of culture. In Geertz C. The interpretation of cultures: Selected essays. New York: Basic Books, pp. 3–30. (1973).

[85] Braun V Clarke V. Thematic analysis: A practical guide. Thousand Oaks, CA: Sage (2021).

[86] Baxter L Montgomery B. Relating: Dialogues and dialectics. New York: Guilford Press (1996).

[87] Toller PW. Negotiation of dialectical contradictions by parents who have experienced the death of a child. J Appl Commun Res. (2005) 33:46–66. doi: 10.1080/0090988042000318512, PMID: 40904582

[88] Hooghe A Neimeyer RA Rober P. The complexity of couple communication in bereavement: an illustrative case study. Death Stud. (2011) 35:905–24. doi: 10.1080/07481187.2011.553335, PMID: 24501858

[89] Ratcliffe M. On feeling unable to continue as oneself. Eur J Philos. (2024) 32:1293–303. doi: 10.1111/ejop.12958

[90] Brison SJ. Aftermath: Violence and the remaking of a self. Princeton, NJ: Princeton University Press (2002).

[91] Humphreys H. True story: On the life and death of my brother. London, UK: Profile Books (2013).

[92] Attig T. How we grieve: Relearning the experience. Oxford: Oxford University Press (2011).

[93] Barboza J Seedall R Hooghe A Kaplow J Bradshaw S. Forming our grief rhythm: the relational window of tolerance for bereaved parents. Fam Process. (2024) 64:13048. doi: 10.1111/famp.13048, PMID: 39142334

[94] Barboza J Seedall R. Coregulatory responsive strategies in bereaved parents. J Loss Trauma. (2025) 1–25:1–25. doi: 10.1080/15325024.2025.2483779, PMID: 40904582

[95] American Psychiatric Association. Diagnostic and statistical manual of mental disorders. 5th ed. Washington, DC: American Psychiatric Press (2022).

[96] Mikulincer M Shaver PR Pereg D. Attachment theory and affect regulation: the dynamics, development, and cognitive consequences of attachment-related strategies. Motiv Emot. (2003) 27:77–102. doi: 10.1023/A:1024515519160

[97] Currier JM Holland JM Neimeyer RA. Sense-making, grief, and the experience of violent loss: toward a mediational model. Death Stud. (2006) 30:403–28. doi: 10.1080/07481180600614351, PMID: 16610156

[98] Worden JW. Grief counseling and grief therapy: A handbook for the mental health practitioner. Berlin: Springer Publishing Company (2018).

[99] Robinaugh DJ Mauro C Bui E Stone L Shah R Wang Y. Yearning and its measurement in complicated grief. J Loss Trauma. (2016) 21:410–20. doi: 10.1080/15325024.2015.1110447

[100] Maciejewski PK Zhang B Block SD Prigerson HG. An empirical examination of the stage theory of grief. JAMA. 297:716. doi: 10.1001/jama.297.7.716, PMID: 17312291

[101] Klass D Silverman PR Nickman SL. Continuing bonds: New understandings of grief. Abingdon: Taylor and Francis (1996).

[102] Black J Belicki K Emberley-Ralph J McCann A. Internalized versus externalized continuing bonds: relations to grief, trauma, attachment, openness to experience, and posttraumatic growth. Death Stud. (2022) 46:399–414. doi: 10.1080/07481187.2020.1737274, PMID: 32175829

[103] Riches G Dawson P. An intimate loneliness, supporting bereaved parents and siblings. Buckingham, UK: Open University. (2002).

[104] Scholtes D Browne M. Internalized and externalized continuing bonds in bereaved parents: their relationship with grief intensity and personal growth. Death Stud. (2015) 39:75–83. doi: 10.1080/07481187.2014.890680, PMID: 25103397

[105] Burke LA Neimeyer RA. Complicated spiritual grief I: relation to complicated grief symptomatology following violent death bereavement. Death Stud. (2014) 38:259–67. doi: 10.1080/07481187.2013.829372, PMID: 24524589

[106] Field NP Nichols C Holen A Horowitz MJ. The relation of continuing attachment to adjustment in conjugal bereavement. J Consult Clin Psychol. (1999) 67:212–8. doi: 10.1037//0022-006x.67.2.212, PMID: 10224731

[107] Field NP. Continuing bonds in adaptation to bereavement: introduction. Death Stud. (2006) 30:709–14. doi: 10.1080/07481180600848090, PMID: 16972368

[108] Field NP Packman JD Ronen R Pries A Davies B Kramer R. Type of continuing bonds expression and its comforting versus distressing nature: implications for adjustment among bereaved mothers. Death Stud. (2013) 37:889–912. doi: 10.1080/07481187.2012.692458, PMID: 24517520

[109] Field NP Gao B Paderna L. Continuing bonds in bereavement: an attachment theory based perspective. Death Stud. (2005) 29:277–99. doi: 10.1080/07481180590923689, PMID: 15849880

[110] Laurie A Neimeyer RA. African Americans in bereavement: grief as a function of ethnicity. OMEGA. (2008) 57:173–93. doi: 10.2190/OM.57.2.d, PMID: 18680889

[111] Neimeyer RA Burke LA Mackay MM Van Dyke Stringer JG. Grief therapy and the reconstruction of meaning: from principles to practice. J Contemp Psychother. (2010) 40:73–83. doi: 10.1007/s10879-009-9135-3

[112] Wortmann JH Park CL. Religion and spirituality in adjustment following bereavement: an integrative review. Death Stud. (2008) 32:703–36. doi: 10.1080/07481180802289507, PMID: 18958959

[113] Braun V Clarke V. Reflecting on reflexive thematic analysis. Qual Res Sport Exerc Health. (2019) 11:589–97. doi: 10.1080/2159676X.2019.1628806

[114] Klass D. Sorrow and solace: neglected areas in bereavement research. Death Stud. (2013) 37:597–616. doi: 10.1080/07481187.2012.673535, PMID: 24520963

[115] Australian Institute of Health and Welfare. Australia’s health 2023: data insights. Canberra: AIHW (2023).

[116] Rosenblatt PC. Grief across cultures: a review and research agenda In: Stroebe MS Hansson RO Schut H Stroebe W, editors. Handbook of bereavement research and practice: Advances in theory and intervention. Washington, DC: American Psychological Association (2008). 207–22.

[117] Valentine C. Bereavement narratives: continuing bonds in the twenty-first century. Abingdon: Routledge, (2008).

