# Supplementary material for: Reciprocal regulation in social support interactions between bereaved parents and their potential supporters: a qualitative study
Source: Front Public Health. 2025 Oct 21;13:1659628. doi: 10.3389/fpubh.2025.1659628 (PMC12613046; doi:10.3389/fpubh.2025.1659628)
Supplement: Supplementary file 1 [file Table_1.DOCX]

**Supplementary Materials**

**NVivo Coding Log**

| Name | Description | Files | References |
| --- | --- | --- | --- |
| Bereaved Parent Recommendations | Psychoeducation - Developmental Stages; Grief is an expression of love. Because you will always love the child, you will always grieve them. Level of grief hasn’t changed, the parent has changed around the grief. | 5 | 13 |
| Experience of Bereaved Parents |  | 0 | 0 |
| Existential Collapse |  | 0 | 0 |
| Affective Collapse | Loss of emotional vitality and desire to engage with life; persistent emotional exhaustion and disinterest. | 10 | 31 |
| Allowing grief to consume you |  | 1 | 1 |
| Anger at What's Happened |  | 10 | 20 |
| at mercy of grief |  | 1 | 2 |
| Avoidance and Triggers eg shopping centres and festivities | Daily life is shaped by the need to avoid reminders or manage unpredictable grief surges. | 9 | 21 |
| Bad Therapeutic Experiences |  | 7 | 10 |
| Child Loss is the Worst Loss |  | 2 | 2 |
| Close off for protection-cannot talk about child |  | 2 | 3 |
| Cognitive and Functional Impairment | Grief interferes with concentration, memory, and emotional regulation. | 3 | 7 |
| Didn’t get to say goodbye to child - sudden |  | 1 | 1 |
| Disruption to Identity and Worldview-phantom limb | Loss of meaning, coherence, or identity following the child’s death. | 4 | 8 |
| Don’t Expect Anyone To Understand |  | 4 | 5 |
| Don’t know what I need |  | 2 | 2 |
| Don’t want to ask for support |  | 1 | 1 |
| Emotional Numbness | Affective blunting or detachment from emotions, often accompanied by apathy and anhedonia. | 2 | 2 |
| Emptiness and Loss |  | 4 | 7 |
| Existential Disorientation | Loss of meaning and coherence in life; struggling to reconcile the child’s death with a previously held worldview. | 10 | 15 |
| Fatalistic |  | 6 | 6 |
| Feel Isolated |  | 4 | 5 |
| Feeling Alienated |  | 1 | 1 |
| Grief is Lonely |  | 1 | 1 |
| Guilt |  | 5 | 12 |
| Hopelessness and Suicidal Ideation | Thoughts of death, questioning life's value, or difficulty imagining a future without the child. | 5 | 9 |
| Intolerance of Mundane Complaints |  | 5 | 9 |
| Living a new normal you don't want |  | 2 | 2 |
| Logistical and experiential change |  | 2 | 2 |
| Loss of Identity and Self Worth | Profound self-doubt, low self-esteem, and questioning of personal value and role after loss. | 0 | 0 |
| Low Expectations of Support |  | 2 | 3 |
| Maladaptive Cognitions and Hypervigilance | Feelings of guilt, fear, or hypervigilance that are cognitively distorted but persistent. | 8 | 17 |
| Persistent Emotional Anguish-Grief Getting Worse | Unrelenting emotional pain that overwhelms functioning and remains unsoothed. | 5 | 11 |
| Putting on a front - 'I'm fine' |  | 3 | 3 |
| Shock |  | 4 | 6 |
| Social Disconnection and Withdrawal | Grief leads to avoidance of social spaces, increased isolation, and detachment from others. | 5 | 11 |
| Social Withdrawal | Retreat from social interaction due to fear of judgment, feeling misunderstood, or emotional exhaustion. | 1 | 2 |
| Stigma and Isolation | Perceived inability of others to sit with grief long-term; fear of being a burden or discomforting others. | 2 | 3 |
| Unrelenting Grief | Thoughts of death, questioning life's value, or difficulty imagining a future without the child. | 2 | 5 |
| Integrated Existence |  | 0 | 0 |
| Active Grief as Expression and Agency | Grief is channelled into active processes such as advocacy, creativity, or group involvement, giving it structure and purpose. | 1 | 1 |
| Adaptive Cognitions |  | 4 | 10 |
| Adaptive Functioning and Experiences |  | 6 | 8 |
| Adaptive Identity Reconstruction | Bereaved parents adapt their identity to integrate the loss, often maintaining continuity with who they were while acknowledging transformation. | 6 | 8 |
| Black Humour |  | 1 | 1 |
| Community and Advocacy | Channelling grief into external action, such as grief groups, foundations, or public awareness. | 8 | 29 |
| Continuing Bonds and Active Remembrance - say name | Maintaining a connection with the deceased child through memory, ritual, or symbolic presence. | 11 | 31 |
| Continuing Bonds through Ritual and Symbols | Grief is made manageable through intentional rituals and symbols that sustain a felt connection with the child. | 5 | 6 |
| Creative and Ritualised Expression | Using art, ritual, humour, or symbolic acts to honour the child and give expression to grief. | 4 | 9 |
| Do it Anyway - Keep functioning |  | 1 | 1 |
| Enduring Grief | Grief remains a persistent presence in the bereaved parent's life, not something to 'get over' but something to live with. | 10 | 17 |
| Eternal Psychological or Spiritual Bond with Child | The parent maintains a psychological or spiritual connection with the child across time. | 0 | 0 |
| Fluctuating Presence of Grief | Grief is sometimes manageable and sometimes overwhelming, appearing and receding unpredictably. | 9 | 24 |
| Get on with it |  | 2 | 2 |
| Got to say goodbye to child |  | 1 | 1 |
| Grief as Love | Grief is understood as a reflection of enduring love; because the love for the child continues, so does the grief. | 3 | 3 |
| Grief Remains the Same, You Change |  | 5 | 6 |
| Grief Work |  | 5 | 10 |
| Growth and Transformation - compassionate | The experience of becoming more compassionate, accepting, or resilient following the loss. | 7 | 13 |
| Holding Dual Realities - Feel Child In Heart | Living with both the presence of grief and the continuation of life; balancing sorrow with moments of meaning or joy. | 5 | 5 |
| Hope and Strength |  | 2 | 2 |
| Integration of Grief into Daily Life | Grief becomes part of the bereaved parent’s daily existence; life is built around it rather than beyond it. | 2 | 2 |
| It's Your Own Journey |  | 2 | 4 |
| New traditions |  | 4 | 5 |
| Open conversation about experience-transparent |  | 2 | 4 |
| Peer Understanding |  | 7 | 16 |
| Physical or Psychological Therapy |  | 2 | 2 |
| Relational Connection to Child Through Others-their friends | Connection is sustained through relationships with the child’s peers or shared commemorative activities. | 4 | 6 |
| Seeking professional support |  | 5 | 9 |
| Speaking about Child |  | 2 | 3 |
| Spiritual and Philosophical Integration | Engagement with spiritual beliefs, existential questions, or philosophies to find coherence or peace. | 14 | 42 |
| Spiritual or Symbolic Connection | Perceived signs or metaphysical experiences offer comfort and reinforce a sense of ongoing bond. | 4 | 8 |
| Time |  | 3 | 5 |
| Understanding of Support Providers Situation-Not Knowing or Different Needs-Acceptance |  | 9 | 26 |
| Unshackled from social expectations |  | 1 | 1 |
| Want to Feel The Pain of Grief |  | 1 | 1 |
| Perceived Experience of Support Providers |  | 0 | 0 |
| Attuned to Grief |  | 0 | 0 |
| Able to Sit With Discomfort | Support providers are emotionally present and can tolerate the rawness of grief without trying to fix or avoid it. | 1 | 1 |
| Authenticity and Emotional Honesty | Supporters are open about not knowing what to say but express care and presence genuinely. | 1 | 2 |
| Compassion Without Pressure | Supporters provide space for grief without imposing timelines, expectations, or positivity. | 1 | 2 |
| Knowing your own limits of giving |  | 1 | 1 |
| Peer Understanding | Support is most effective when offered by others who have experienced similar loss and understand its depth. | 7 | 12 |
| Speak about Child and Keep Them Alive |  | 2 | 2 |
| Misattuned to Grief |  | 0 | 0 |
| Blame |  | 2 | 3 |
| Discomfort and Emotional Shutdown - dont know what to say | Supporters display awkwardness, panic, or say nothing out of fear of saying the wrong thing. | 6 | 8 |
| Emotional Misattunement | Supporters offer advice, cheerfulness, or distractions that invalidate or minimise grief. | 2 | 2 |
| Fear and Avoidance | Supporters are overwhelmed by the intensity of grief and withdraw or change the subject. | 4 | 5 |
| Lack of Understanding |  | 6 | 11 |
| Over-personalising and Centring on Self | Supporters shift the focus to their own discomfort, spiritual beliefs, or unrelated experiences. | 3 | 4 |
| Support Interactions |  | 0 | 0 |
| Interactions |  | 0 | 0 |
| Helpful |  | 0 | 0 |
| Active Support |  | 7 | 11 |
| Attuned Presence - Compassion - Listen and Let Parent Talk-Acceptance | Supporters who offer presence, silence, and emotional safety without trying to fix or avoid the grief. | 14 | 51 |
| Availability and Reliability and Consistency |  | 7 | 12 |
| Conversations that ebb and flow about child |  | 2 | 4 |
| Dont feel judged |  | 1 | 1 |
| Empathy |  | 1 | 1 |
| Gentle touch and no words |  | 6 | 9 |
| Give hope and strength |  | 1 | 3 |
| Interacting with Child's Friends |  | 3 | 6 |
| Kind Communication |  | 2 | 2 |
| Letting the Parent Lead | Being guided by the bereaved parent’s emotional cues, offering flexibility and sensitivity. | 4 | 4 |
| Little Sensitive Things e.g. poem |  | 2 | 4 |
| Mutual Understanding and Trust |  | 3 | 3 |
| Normalise Grief Response |  | 1 | 1 |
| Open Conversations |  | 2 | 2 |
| Open straightforward no sugar coating and honest |  | 4 | 8 |
| Other Bereaved Parents Get It |  | 12 | 35 |
| People in outer circles are supportive |  | 1 | 3 |
| Reaching Out Unprompted | Supporters who check in, send messages, or acknowledge key dates without being asked. | 7 | 10 |
| Say Childs Name and Honour Them-keep them alive |  | 9 | 21 |
| Supporter Open to Learning about Grief |  | 2 | 2 |
| Trust Supporter and Feel Safe-known to parent and they knew child |  | 8 | 17 |
| Unconditional Support |  | 3 | 3 |
| Understanding |  | 8 | 17 |
| Validation and Permission and Acknowledgement | Acknowledging the pain, allowing space to cry, and reassuring that emotions are acceptable. | 6 | 10 |
| Unhelpful |  | 0 | 0 |
| Avoidance and Withdrawal-Don’t know what to say | Supporters who disappear, avoid contact, or change the subject when grief arises. | 14 | 62 |
| Different Needs |  | 7 | 10 |
| Disempowered |  | 1 | 2 |
| Don't want sympathy or pity |  | 3 | 3 |
| Emotional Incompetence and Disappointment | Supporters who try to fix the grief, give advice, or make the conversation about themselves. | 10 | 38 |
| Expect parent to reach out to supporter |  | 3 | 4 |
| Hypocrisy |  | 1 | 1 |
| Inappropriate Questions and Advice |  | 5 | 12 |
| Insensitive with no understanding of needs |  | 7 | 8 |
| It will get better, Move On |  | 5 | 5 |
| Judgement, no understanding and Dismissal | Comments that minimise, question, or judge the parent’s way of grieving. | 10 | 23 |
| Only there for the drama then disappears |  | 1 | 3 |
| Prioritising Religion When Parent Not Religious |  | 3 | 8 |
| Rumours |  | 1 | 1 |
| Strangers |  | 1 | 1 |
| Supporter is fearful |  | 1 | 1 |
| Supporter making it About Themselves |  | 9 | 29 |
| Supporter Uncomfortable and cannot talk about child |  | 7 | 14 |
| Talking too Much - Mere platitudes |  | 6 | 9 |
| Unreliable - Unpredictable Support |  | 2 | 3 |
| Reciprocal Regulation |  | 0 | 0 |
| Co-regulation Through Relational Presence | Emotional regulation facilitated through relational presence and attunement. | 4 | 8 |
| Distance myself because own mental health at risk |  | 4 | 7 |
| Educate supporters |  | 2 | 4 |
| Emotional Synchrony through Mirror Neuron Resonance | Emotional synchrony through facial expression, tone, and presence that fosters mutual understanding. | 1 | 1 |
| Frustration |  | 2 | 2 |
| Mutual Relational Transparency | Openness and honesty from supporters (e.g., admitting discomfort) fosters social safety. | 5 | 10 |
| Recalibration of Social Network peripheral network replace inner | Realignment of social relationships post-loss based on emotional capacity and presence. | 12 | 47 |
| Strengthened Dyad - Safety and Attachment | Trust and connection deepen through attuned support. | 6 | 16 |
| Weakened Dyad - Reciprocal Avoidance | Avoidance, overstepping, or insensitivity disrupts the relationship. | 10 | 40 |
| Reciprocity in Emotional Support - It Takes Two | Shared emotional labour; both parties contribute to the tone and safety of the interaction. | 6 | 12 |
| Shock Supporter Into Awareness |  | 5 | 7 |
| Withdrawal from Misattunement-no expectations | Emotional retreat triggered by being misunderstood, judged, or dismissed. | 11 | 14 |
| Societal Factors (Western Norms) |  | 0 | 0 |
| Assumed Support from Family and Community | Assumption that adequate support is already provided by others, leading to less outreach. | 1 | 2 |
| Conform to Societal Expectations |  | 6 | 10 |
| Death Denial and Death Anxiety (Discomfort) | A widespread discomfort with the topic of death, leading to silence or euphemisms. | 11 | 31 |
| Discomfort with Emotional Expression | Cultural pressure to maintain composure and avoid vulnerability in public. | 3 | 8 |
| Get on with it |  | 6 | 9 |
| Intractable Problem |  | 2 | 3 |
| Little Support for parents in community |  | 2 | 2 |
| Loss of Shared Rituals or Frameworks | Modern society lacks meaningful rituals or frameworks for supporting grief. | 0 | 0 |
| Make new Traditions e.g. bring back black armband |  | 1 | 1 |
| Misuse of Grief Models and Shoulds | Societal expectations that impose a linear or appropriate way to grieve (e.g., ‘move on,’ ‘stay strong’). | 2 | 2 |
| Need Open Conversations-Psychoeducation |  | 2 | 4 |
| No understanding |  | 3 | 3 |
| Only Other Bereaved Parents Understand |  | 3 | 4 |
| Pressure to Distract or Fix Bereaved Parent | Supporters often default to offering distractions or superficial reassurance rather than bearing witness | 4 | 4 |
| Stigma of Certain Types of Death | Suicide, overdose, or traumatic deaths are often stigmatised, leading to silence or social withdrawal. | 7 | 11 |
| Surface Level Functioning - Productivity, Positivity |  | 1 | 7 |
| Time Limit on Grief |  | 8 | 12 |
| Unrealistic Expectations |  | 1 | 2 |
| Want parent back to normal-not hurting |  | 3 | 5 |

**SRQR Compliance Table**

| **SRQR Item** | **Description** | **Addressed** | **Location in Manuscript** | **Notes / Gaps** |
| --- | --- | --- | --- | --- |
| **1. Title** | Concise description of study and qualitative nature | Yes. | Title page | Includes focus on bereaved parents, qualitative approach |
| **2. Abstract** | Structured summary of key elements | Yes. | Abstract | Could add explicit mention of constructivist–interpretivist paradigm |
| **3. Problem formulation** | Problem, significance, literature review | Yes. | Introduction | Links to literature on Western norms, grief avoidance, and social support |
| **4. Purpose / research question** | Specific objectives or research question | Yes. | Introduction | Clearly stated: exploring lived experiences and support interactions post-child loss |
| **5. Qualitative approach and research paradigm** | Approach and paradigm | Yes. | Methods – Theoretical Framework | Reflexive thematic analysis (Braun & Clarke, 2022), constructivist–interpretivist paradigm |
| **6. Researcher characteristics and reflexivity** | Researcher background, positioning, reflexivity strategies | Yes. | Methods – Researcher Characteristics and Reflexivity | Includes credentials, clinical background, reflexive journaling, peer debriefing |
| **7. Context** | Setting and relevant contextual factors | Yes. | Methods – Setting | Australian context, Western grief norms, interviews conducted online, phone, or in person |
| **8. Sampling strategy** | How and why participants were selected | Yes. | Methods – Sampling | Purposive sampling of bereaved parents with diversity of experiences |
| **9. Ethical issues** | IRB/ethics approval, consent | Yes. | Methods – Ethics | Approved by Curtin University HREC; written informed consent obtained |
| **10. Data collection methods** | Procedures, changes over course of study | Yes. | Methods – Data Collection | Semi-structured interviews, minor wording changes after early interviews |
| **11. Data collection instruments & technologies** | Interview guide, recording methods | Yes. | Methods – Data Collection | Semi-structured guide, audio-recording, secure transcription |
| **12. Units of study** | Number and characteristics of participants | Yes. | Methods – Participants | 16 bereaved parents, varied causes of child death, demographics |
| **13. Data processing** | How data were prepared for analysis | Yes. | Methods – Data Analysis | Verbatim transcription, de-identification, NVivo management |
| **14. Data analysis** | Analytic process, researchers involved | Yes. | Methods – Data Analysis | Reflexive thematic analysis, iterative coding, peer review |
| **15. Techniques to enhance trustworthiness** | Credibility, dependability, confirmability, transferability | Yes. | Methods – Rigour and Trustworthiness | Reflexivity, member checking, peer debriefing, audit trail |
| **16. Synthesis and interpretation** | Main analytic outcomes | Yes. | Results | Four themes and eight subthemes developed |
| **17. Links to empirical data** | Use of participant quotes, field notes | Yes. | Results | Rich quotes supporting each theme |
| **18. Integration with prior work** | Compare with prior studies | Yes. | Discussion | Linked to grief and social support literature |
| **19. Limitations** | Limitations of study and trustworthiness | Yes. | Discussion – Limitations | Includes sample size, representativeness, and lack of triangulation |
| **20. Implications** | Practice, policy, and future research | Yes. | Conclusion | Emphasis on emotional literacy, support training, and policy change |
| **21. Funding** | Source and role of funders | Yes. | Acknowledgements | No funder influence |

**COREQ Compliance Table**

| **COREQ Item** | **Description** | **Addressed** | **Location in Manuscript** | **Notes / Gaps** |
| --- | --- | --- | --- | --- |
| **Domain 1: Research Team & Reflexivity** |  |  |  |  |
| **1. Interviewer/facilitator** | Who conducted the interviews | Yes. | Methods – Researcher Characteristics | JT conducted all interviews |
| **2. Credentials** | Researchers’ credentials | Yes. | Methods – Researcher Characteristics | JT is a registered psychologist and PhD candidate. LB has 20+ years of grief research. DR has research expertise in cognitive and emotional processes. |
| **3. Occupation** | Occupation at time | Yes. | Methods – Researcher Characteristics | JT is a psychologist & researcher. LB and DR are academics. |
| **4. Gender** | Researchers’ genders | Yes. | Methods – Researcher Characteristics | JT and LB are female. DR is male. |
| **5. Experience/training** | Training relevant to research | Yes. | Methods – Researcher Characteristics | JT has 5 years’ bereavement counselling/research experience. LB has a PhD and 20+ years of research experience. DR has a PhD and 10+ years of research experience. |
| **6. Relationship established** | Pre-existing relationship? | Yes. | Methods – Researcher Characteristics | None prior to recruitment |
| **7. Participant knowledge of researcher** | What participants knew | Yes. | Methods – Researcher Characteristics | Informed about professional background, aims |
| **8. Interviewer characteristics** | Bias, assumptions, interests | Yes. | Methods – Reflexivity | Reflexive journaling, consultation among the team |
| **Domain 2: Study Design** |  |  |  |  |
| **9. Methodological orientation** | Theory underpinning study | Yes. | Methods – Theoretical Framework | Constructivist–interpretivist, reflexive TA |
| **10. Sampling** | Sampling method | Yes. | Methods – Sampling | Purposive sampling |
| **11. Method of approach** | Recruitment method | Yes. | Methods – Sampling | Email via bereavement support networks |
| **12. Sample size** | Number of participants | Yes. | Methods – Participants | 16 |
| **13. Non-participation** | Refusals/dropouts | Partial. | Methods – Sampling | Not all reasons for non-participation documented |
| **14. Setting of data collection** | Where interviews took place | Yes. | Methods – Setting | Participant homes, online |
| **15. Presence of non-participants** | Others present? | Yes. | Methods – Setting | None |
| **16. Description of sample** | Key characteristics | Yes. | Methods – Participants | Demographic & experiential variation |
| **17. Interview guide** | Use of guide/pilot | Yes. | Methods – Data Collection | Semi-structured guide |
| **18. Repeat interviews** | Were they conducted? | Yes. | Methods – Data Collection | No repeat interviews |
| **19. Audio/visual recording** | Recording method | Yes. | Methods – Data Collection | Audio recording |
| **20. Field notes** | Field notes taken? | Yes. | Methods – Data Collection | Reflective field notes post-interview |
| **21. Duration** | Length of interviews | Yes. | Methods – Data Collection | 60–120 minutes |
| **22. Data saturation** | Addressed? | Yes. | Methods – Data Collection | Information power |
| **23. Transcripts returned** | Member checking? | Yes. | Rigour & Trustworthiness | Thematic summaries returned; 7 participants provided feedback |
| **Domain 3: Analysis & Findings** |  |  |  |  |
| **24. Number of data coders** | Who coded data | Yes. | Methods – Data Analysis | JT coded; LB and DR reviewed |
| **25. Description of coding tree** | Coding framework | Yes. | Methods – Data Analysis | Full coding map provided |
| **26. Derivation of themes** | How themes identified | Yes. | Methods – Data Analysis | Inductive coding, iterative refinement |
| **27. Software** | Software used | Yes. | Methods – Data Analysis | NVivo |
| **28. Participant checking** | Validation of findings | Yes. | Rigour & Trustworthiness | Member checking of thematic summaries |
| **29. Quotations presented** | Participant quotes | Yes. | Results | Rich, varied quotes |
| **30. Data and findings consistent** | Coherence between quotes and themes | Yes. | Results | Clear mapping |
| **31. Clarity of major themes** | Themes clearly presented | Yes. | Results | Four themes, eight subthemes |
| **32. Clarity of minor themes** | Minor/divergent cases reported | Yes. | Results | Deviant cases noted in text |

**Braun and Clarke’s 15-point checklist for good reflexive thematic analysis.**

|  | Process | Criteria | Response |
| --- | --- | --- | --- |
| 1 | **Transcription** | The data have been transcribed to an appropriate level detail; all transcripts have been checked against the recordings for ‘accuracy’. | Yes. All transcripts were checked against the recordings for accuracy to ensure authentic records for analysis. |
| 2 | **Coding** | Each data item has been given equal attention in the coding process. | Yes. The first author reviewed the entire data corpus of quotations to generate coding. |
| 3 |  | Themes have not been generated from a few vivid examples (an anecdotal approach), but instead the coding process has been thorough, inclusive and comprehensive. | Yes. Themes, and the findings described herein, were developed from a complete coding process of the entire dataset. The coding process was thorough, inclusive and comprehensive, as all quotations were used to generate codes, and develop themes. Each theme was developed based on numerous codes gathered across a range of articles and participants quotations. |
| 4 |  | All relevant extracts for each theme have been collated. | Yes. The manuscript includes multiple examples of this. |
| 5 |  | Themes have been checked against each other and back to the original data set. | Yes. The first author ensured coherence in process and reporting. |
| 6 |  | Themes are internally coherent, consistent, and distinctive. | Yes. Research team members reviewed interim themes to ensure themes are internally coherent, consistent, distinctive and centred on an organising concept. |
| 7 | **Analysis** | Data have been *analysed* – interpreted, made sense of – rather than just paraphrased or described. | Yes. Evident from the results (i.e., level of interpretation rather than description). |
| 8 |  | Analysis and data match each other – the extracts evidence the analytic claims. | Yes. The analysis and findings from it closely match the data set. |
| 9 |  | Analysis tells a convincing and well-organised of story about the data and topic; analysis addresses the research question. | Yes. Analysis led to the development of 4 organised themes that address the research aim. |
| 10 |  | A good balance between analytical narrative and illustrative extracts is provided. | Yes. Illustrative extracts have been used within the results section. |
| 11 | **Overall** | Enough time has been allocated to complete all phases of the analysis adequately, without rushing a phase or giving it a once-over-lightly | Yes. |
| 12 | **Written report** | The assumptions about, and specific approach to, thematic analysis are clearly explicated. | Yes. Stated in the methods section. |
| 13 |  | There is a good fit between what you claim you do, and what you show you have done, i.e., the described method and reported analysis are consistent. | Yes. The description of the research methods, analytical processes, and interpretations is consistent. |
| 14 |  | The language and concepts used in the report are consistent with the epistemological position of the analysis | Yes. |
| 15 |  | The researcher is positioned as active in the research process; themes do not just ‘emerge’. | Yes. |
